# Acitretin reverses early functional network degradation in a mouse model of familial Alzheimer’s disease

**DOI:** 10.1038/s41598-021-85912-0

**Published:** 2021-03-23

**Authors:** Eduardo Rosales Jubal, Miriam Schwalm, Malena dos Santos Guilherme, Florian Schuck, Sven Reinhardt, Amanda Tose, Zeke Barger, Mona K. Roesler, Nicolas Ruffini, Anna Wierczeiko, Michael J. Schmeisser, Ulrich Schmitt, Kristina Endres, Albrecht Stroh

**Affiliations:** 1 Focus Program Translational Neurosciences, University Medical Center of the Johannes Gutenberg-University, Mainz, Germany; 2 Institute for Pathophysiology, University Medical Center of the Johannes Gutenberg University Mainz, Hanns-Dieter-Hüsch-Weg 19, 55128 Mainz, Germany; 3 Competence Center for Methodology and Statistics, Luxembourg Institute of Health, 1A-B, rue Thomas Edison, 1445 Strassen, Luxembourg; 4 GRADE Brain, Goethe Graduate Academy, Goethe University Frankfurt Am Main, Frankfurt am Main, Germany; 5 Department of Biological Engineering, Massachusetts Institute of Technology, Cambridge, MA USA; 6 Department for Psychiatry and Psychotherapy, University Medical Center of the Johannes Gutenberg-University, Mainz, Germany; 7 Department of Molecular and Cell Biology, Helen Wills Neuroscience Institute, UC, Berkeley, CA USA; 8 Institute for Microscopic Anatomy and Neurobiology, University Medical Center of the Johannes Gutenberg-University, Mainz, Germany; 9 Leibniz Institute for Resilience Research, Mainz, Germany

**Keywords:** Neuroscience, Neural ageing, Neural circuits, Neuronal physiology, Synaptic plasticity, Visual system

## Abstract

Aberrant activity of local functional networks underlies memory and cognition deficits in Alzheimer’s disease (AD). Hyperactivity was observed in microcircuits of mice AD-models showing plaques, and also recently in early stage AD mutants prior to amyloid deposition. However, early functional effects of AD on cortical microcircuits remain unresolved. Using two-photon calcium imaging, we found altered temporal distributions (burstiness) in the spontaneous activity of layer II/III visual cortex neurons, in a mouse model of familial Alzheimer’s disease (5xFAD), before plaque formation. Graph theory (GT) measures revealed a distinct network topology of 5xFAD microcircuits, as compared to healthy controls, suggesting degradation of parameters related to network robustness. After treatment with acitretin, we observed a re-balancing of those network measures in 5xFAD mice; particularly in the mean degree distribution, related to network development and resilience, and post-treatment values resembled those of age-matched controls. Further, behavioral deficits, and the increase of excitatory synapse numbers in layer II/III were reversed after treatment. GT is widely applied for whole-brain network analysis in human neuroimaging, we here demonstrate the translational value of GT as a multi-level tool, to probe networks at different levels in order to assess treatments, explore mechanisms, and contribute to early diagnosis.

## Introduction

Alzheimer’s disease (AD) has devastating effects on years of healthy life. Interventions that could delay disease onset even modestly would have a major public health impact^1^ as there is only symptomatic medication available that merely results in minor improvements of cognitive abilities^2^, while curative or even preventive drugs are lacking. Therapeutic strategies identified in preclinical experiments have failed probably due to recruitment of patients who already displayed manifested AD^3^. Indeed, a hallmark of AD is the subtle pre-clinical progression^4^, hindering diagnosis in early stages.

A characteristic finding in affected brain regions of patients are extracellular depositions of amyloid beta (Aβ) in the form of plaques^5^. However, the amount and distribution of Aβ deposition seem to be only weakly correlated with clinical AD stage, despite the involvement of Aβ in inducing synaptic dysfunction and association with region-specific neuronal death^6^. Indeed, the most robust correlation in the staging of dementia and early AD is the magnitude of synapse loss^7^, while non-fibrillar soluble forms of Aβ seem to be mediators of synaptic decline, leading to neurodegenerative effects^8^. Accumulation of Aβ begins several decades before AD symptom onset in human patients^9^. Asymptomatic individuals with cerebral amyloidosis have an increased risk of subsequent cognitive decline, including development of AD dementia^10^, although lacking obvious structural abnormalities^11^. The subsequent cognitive decline might be a consequence of disruptions in the structural and functional connections in local and global networks^11^.

Neuronal networks in animal models of AD^12–16^ exhibit hyperactivity in calcium imaging and electrophysiological measures^12,16,17^, representing a hallmark of local AD network dynamics. Hyperactivity was both identified in the vicinity of plaques at later stages of AD^12^, as well as in early stages prior to plaque deposition^18^. Notably, hyperactivity is also observed in the brain of human AD patients^19^. However, while cellular hyperactivity seems to be a common factor in early stages of neurodegenerative and also neuroimmunological disorders^18,20,21^, the effect on higher-order network topology remains unknown. Hyperactivity refers to enhanced firing rate at single cell level, but does not inform about the distribution of those firing patterns. Spikes organized in groups are known as bursts, characterized by short intervals between individual events (inter-event intervals, IEI). Bursting firing pattern can be understood as an interaction between lifetime sparseness^22^, i.e. the spike rate of an individual neuron, and the distribution of such firing events. Both, simple firing rate and burstiness analyses, are performed at the single-cell level, and thus do not shed light on emerging network dynamics.

Graph theory is widely used to study biological networks^23–27^, because it allows to describe a network’s stability, connectivity, and organization through a number of parameters^28,29^. Since the brain's structural and functional systems can be understood as complex networks^23^, this approach has been rapidly translated to quantitative analysis of brain network organization with a vast number of studies investigating human AD imaging data^11,30–33^. In those studies, whole-brain network topology was shown to be disrupted in fMRI networks affecting their global efficiency and modularity – two of many parameters used for network topology assessment—in late^34^ as well as in early stages of AD^11,35^.

Two-photon imaging of local cortical microcircuits using fluorescent calcium indicators monitors optical correlates of activity-related somatic increase in intracellular calcium in single neurons^36^. This approach can detect small, subtle dysregulations, such as hyperactivity within microcircuits, employing the visual cortex as a model region of cortical dysfunction across disorders^20,21^. Nevertheless, monitoring each neuron’s activity over time provides limited information regarding the network’s interactions and topology. Moving beyond a single-neuron based assessment of microcircuits, employing higher-order network analyses such as graph theory, would inform about such measures while preserving the sensitivity provided by imaging a small neuronal assembly, treating single neurons as network nodes. The graph theoretical approach allows to identify network instability within in vivo cortical microcircuits, probing the influence of individual neurons exhibiting aberrant activity such as burstiness, on the vulnerability of a local neuronal network. Additionally, these measures can be used to assess effects of treatments aiming at network restoration.

Here, we investigated ongoing activity of neurons in the visual cortex (V1) of a genetic mouse model of AD carrying the mutated human amyloid precursor protein (APP) gene as well as human mutated presenilin 1 (5xFAD, APP K670N, M671L, I716V; PS1 M146L,L286V;^37^), prior to the onset of apparent neurodegeneration and pronounced plaque formation. We identify burstiness characterized by reduced IEIs of the calcium traces in a small portion of neurons within V1. We chose to conduct the study in a controlled lightly anesthetized condition, as in previous studies of ours and others^12,20,21^ to reduce both, physiological noise and the impact of behavioral state. Assessing network topology by treating individual neurons as nodes revealed a significant degradation of parameters related to the network’s robustness and its capability to compensate node loss, suggesting network vulnerability even before the onset of plaque formation. We compare network topology measures in 5xFAD and wild type (WT) mice, as well as in age-matched 5xFAD mice after treatment with the synthetic retinoid acitretin, a promising AD drug subject to extensive pre-clinical testing^38–41^. We provide evidence that acitretin stabilizes cortical microcircuit dynamics and rebalances network topology parameters related to network development and resilience in early-stage 5xFAD mice. Acitretin treatment also normalized aberrant synapse numbers, and ameliorated behavioral deficits. Leveraging information about interactions between neurons provided by two-photon imaging data, we show graph theoretical analysis to inform about microcircuit network topology, thus serving to monitor disease progression and potential restoration.

## Materials and methods

Detailed methods are provided in* SI Materials and Methods.*

### Animals

Animal husbandry and experimental manipulation were carried out according to animal welfare guidelines of the Johannes Gutenberg-University Mainz and were approved by the ethics committee of the Landesuntersuchungsamt Rheinland-Pfalz, Koblenz, Germany. All animals used in this study were 16 week old males. Age-matched wild type littermates were used as controls. All experiments were performed in accordance with the ARRIVE guidelines (http://www.nc3rs.org.uk/page.asp?id=1357).

### Two-photon imaging

A Craniotomy over binocular visual cortex and bulk loading of the fluorescent calcium indicator Oregon Green Bapta-1 AM (OGB-1; 500 μM; Life Technologies/ Molecular Probes, Waltham, USA) were performed as described elsewhere (58). In-vivo imaging was performed with a custom built two-photon microscope based on a Ti:Sapphire laser (Ti:Sa) with 700–1000 nm tunable output, equipped with a resonance scanner, operating at 800 nm wavelength (LaVision Biotec, Bielefeld, Germany) and a 25X (1.1 N.A.; model-nr: MRD77220. Nikon, Tokyo, Japan) water immersion objective. Data analysis was performed off-line using custom-written MATLAB scripts (Mathworks, Nathick, MA, USA).

### Quantification and statistical analysis

We determined the sample size based on previous publications reporting in-vivo calcium imaging in awake and lightly anesthetized mice. Statistical measures were calculated in the parametric space, when normality (Lilliefors-Test) and homoscedasticity criteria allowed it, or with non-parametric statistics otherwise. The specific tests are indicated in the figure legends. Values are reported as mean ± SEM for normally distributed data. When non-parametric statistics were used, mean ranks are reported.

### Acitretin treatment

5xFAD Mice were given a 9 days treatment, consisting of 5 consecutive days of one daily injection, 2 days of rest, 2 days of daily injections. Mice received a dosage of 10 mg/kg in a total volume of 400 µl corn oil via i.p. injection. On day 10, the animals were tested either behaviorally or in two photon calcium imaging.

### Immunohistochemistry

For each genotype/condition 3 slices of 3 animals were analyzed. An additional slice was used as a control and only treated with secondary antibodies (Supplementary Fig. 4). Layers II/III, layer IV and layers V/VI were detected in each slice using DAPI staining. Slices were washed 3 times for 5 min in 1 × PBS before they were incubated in blocking solution (20% BSA, 0.2% Triton in 1 × PBS) for 2 h at RT. The solution was exchanged with fresh blocking solution (10% BSA, = 0.2% Triton in 1 × PBS) including primary antibodies for 48 h. Refer to SI for details on antibodies and concentrations used (Table S4).

### Significance statement

Targeting early network dysregulations prior to irrevocable neurodegeneration might represent a promising therapeutic approach in Alzheimer’s disease (AD). Here, we applied graph theoretical approaches to identify these dysregulations in a mouse model of AD, prior to neurodegeneration and plaque formation. We find a distinct network pathology characterized by a significant degradation of parameters related to the network's robustness and resilience in cortical microcircuits. Notably, upon treatment with the clinically available retinoid acitretin, deterioration of network topology measures, and structural synapse numbers was reversed, alongside an amelioration of behavioral dysfunction. This study may lay the foundation for neurophysiologically-informed, network-centered therapeutic interventions, aiming for stabilizing network function and delaying disease progression.

## Results

### Aβ deposition and behavioural deficits in early-stage 5xFAD mice

**Figure 1:**
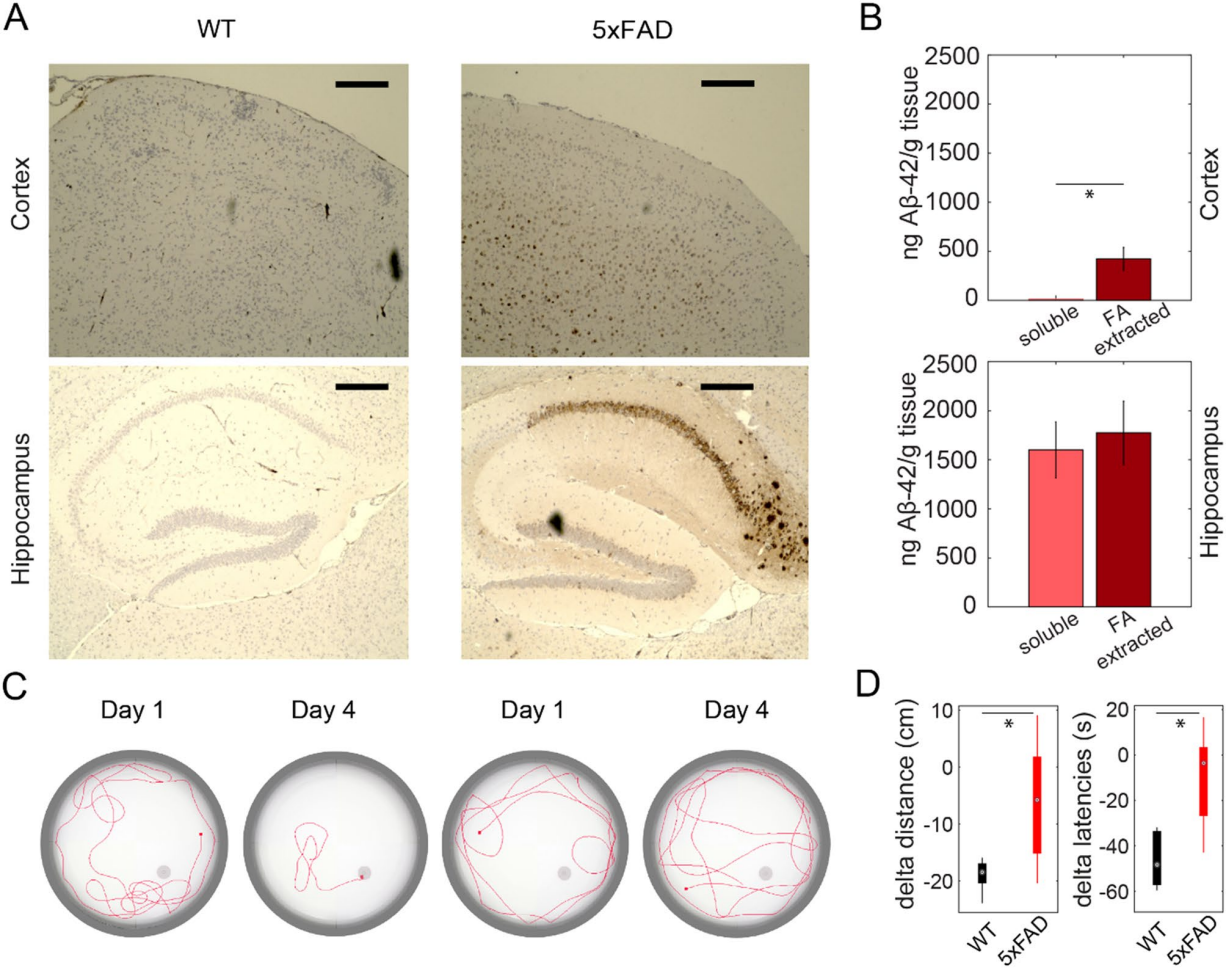
Early-stage 5xFAD mice exhibit early deficits in visuospatial learning but only limited Aβ deposition. (**A**) Analysis of Aβ depositions in 4 month old 5xFAD mice reveals only comparably low levels in cortical tissue. Immunohistochemical analysis for wild type controls (WT, left column) and Alzheimer’s disease model mice (5xFAD, right column); in cortex (top row) and hippocampus (bottom row). Mice of either genotype were sacrificed at four months of age and sagittal slices of the brain stained with antibody 6E10 (scale bar: 250 µm). (**B**) Quantification of Aβ peptides in hippocampus and total cortical tissue shows low level of soluble and plaque-associated (formic acid-extractable, FA) peptides in the cortex, while exhibiting higher levels in hippocampus (n = 4 mice). Obtained values were normalized to tissue wet weight and are given as mean ± SEM (unpaired *t* test; soluble vs. FA-extracted: cortex: t(6) = − 3.5627, *p* = 0.0119, hippocampus: t(6) = − 0.4035, *p* = 0.7006). (**C**) Swim-paths of representative animals of WT (left) and 5xFAD (right) groups on day 1 and 4 of Morris water maze demonstrate 5xFAD mice exhibiting deficits in comparison to WT animals. (**D**) Swimming distance (left) and latencies until reaching a hidden platform (right) are significantly longer for 5xFAD in comparison to WT animals in the Morris water maze (n = 5 mice).

In 5xFAD mice, it has been shown that Aβ depositions first appear in deep layers of the cortex and the subiculum, to later spread towards other cortical regions and hippocampus^37^. We compared Aβ deposition in cortex and hippocampus of 5xFAD mice and wild type littermates (WT) at 4 months of age (Fig. 1A). In cortical tissue, soluble peptides only represented a fractional amount and plaque-associated peptides still did not reach high levels (1.9 ± 0.7 vs 422.6 ± 118.1 ng/g; Fig. 1B, top row). Whereas in hippocampus, soluble and insoluble Aβ_42_ peptides both were detected at higher, similar levels (Fig. 1B, bottom row; *p* = 0.0113 for comparison of soluble A-beta in both tissue specimen; *p* = 0.0077 for comparison of Formic Acid (FA) extracted A-beta in both tissue specimen).

At the same age, transgenic mice show deficits in visuospatial learning and memory. In the Morris Water Maze task (MWM;^42^) 5xFAD mice performed significantly worse than WT controls regarding latency (5xFAD = − 10.5 ± 10.1, WT = − 46.1 ± 5.6; t(8) = − 3.0735, *p* = 0.0153) and traveled distance (5xFAD = − 6.2 ± 5.1, WT = − 18.9 ± 1.3; t(8) = − 2.4107, *p* = 0.0425) for finding the submerged platform (Fig. 1C,D).

These results are consistent with the fact that first signs of memory loss are found at 4 months of age in 5xFAD mice (e.g.,^43^). However, these animals are still unimpaired in sensory-motor abilities, and it known that neuronal loss occurs much later^44^. These findings led us to choose 4 months as a model of early-stage AD in 5xFAD mice for analyzing early changes in visual cortex network dynamics and their potential restoration by acitretin treatment.

### 5xFAD mice display aberrant temporal distribution of calcium transients in a small portion of visual cortex

**Figure 2:**
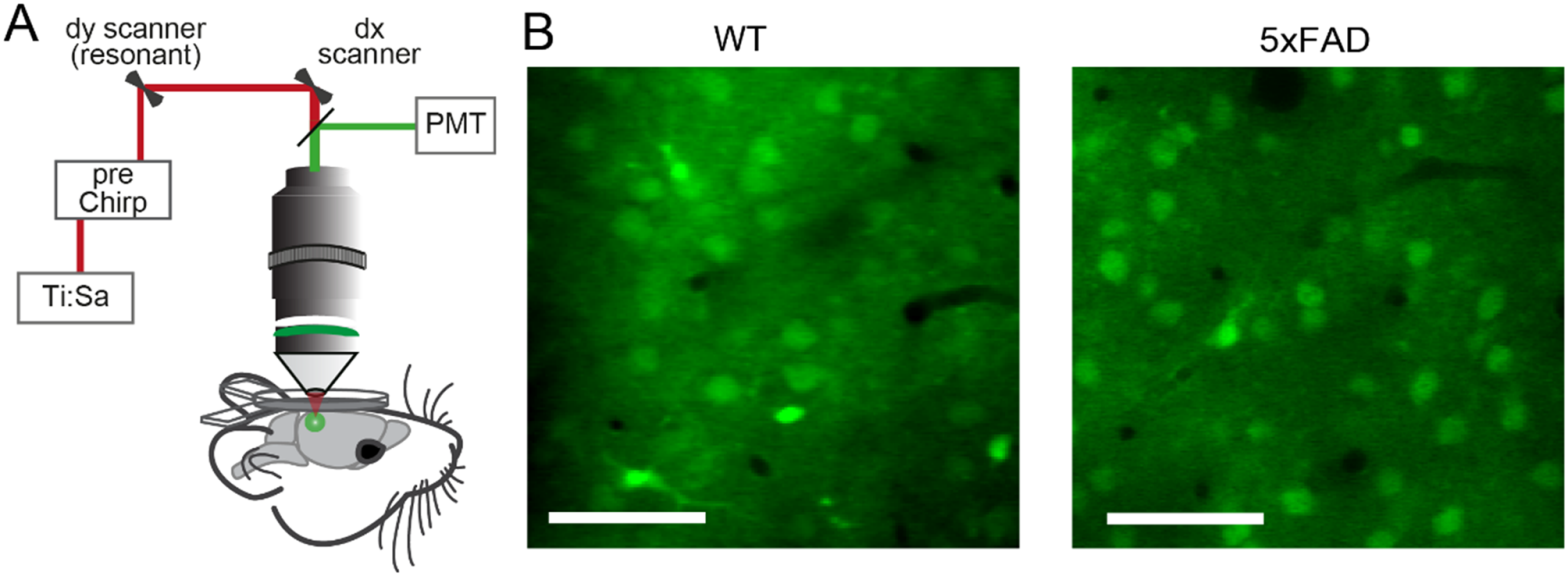
Two-photon imaging in WT and 5xFAD mice. (**A**) Schematic of in vivo two-photon microscopy preparation (not to scale). (**B**) Two-photon images of OGB-1 AM staining in layer II/III of V1 in WT (left) and 5xFAD mice (right) showing neuronal somata and astrocytes distinguishable by their endfeet. Note that plaque formations would be visible by OGB-1 staining (60). Scale bars: 50 µm.

**Figure 3:**
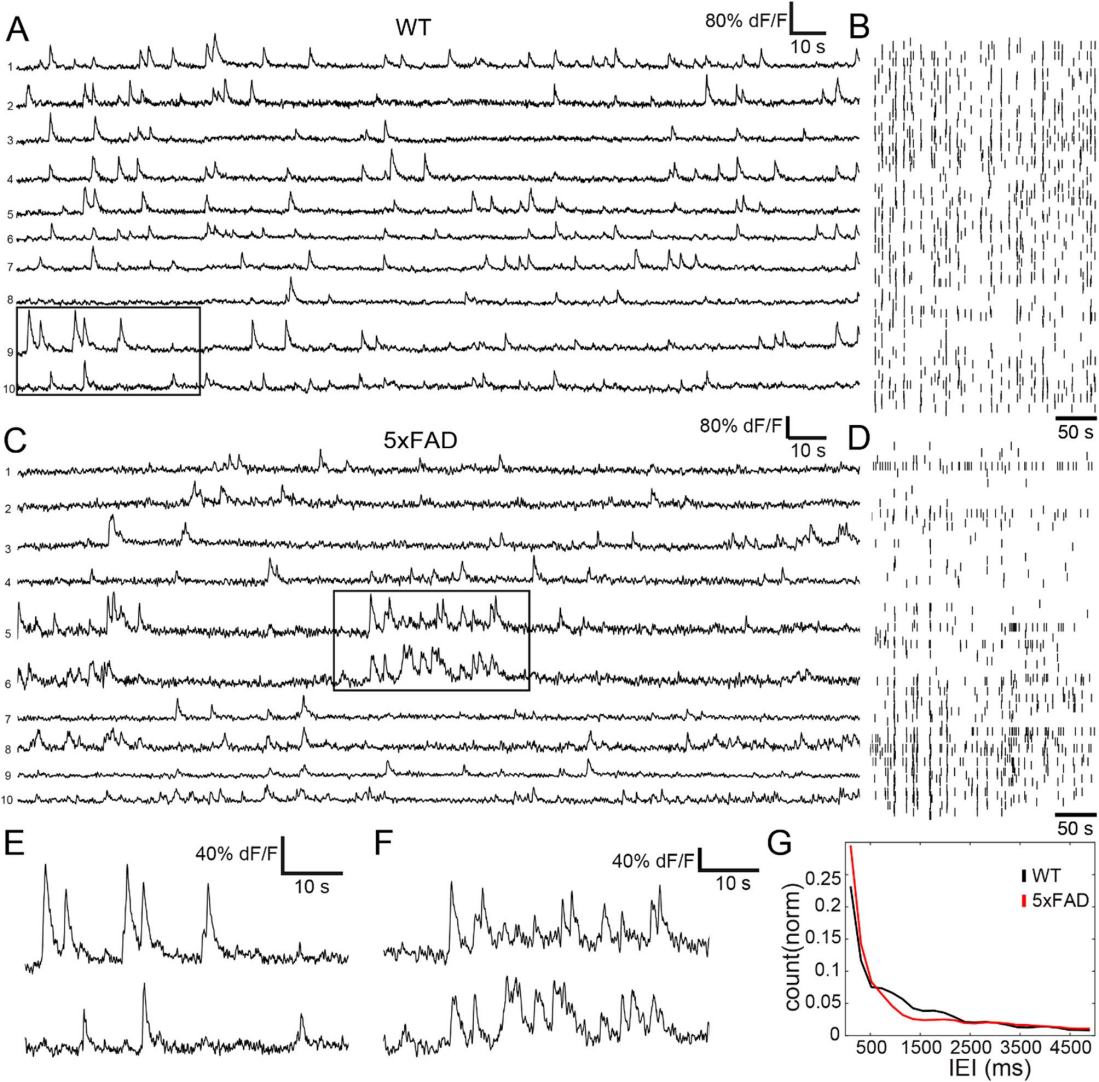
5xFAD model exhibits aberrant single-cell activity in local microcircuits of V1 in comparison to WT mice. (**A**) Representative traces of calcium transients of 10 neurons from WT control group; ongoing activity in layer II/III neuronal assemblies in V1 cortex. (**B**) Raster plot from entire cell population from which selection in (**A**) was drawn shows calcium transient pattern across whole imaging session. (**C**) Representative traces of 10 neurons from 5xFAD group; ongoing activity in layer II/III neuronal assemblies in V1 cortex. (**D**) Raster plot from entire cell population from which selection in (**C**) was drawn shows calcium transient pattern across whole imaging session. (**E**). Magnification of section highlighted in (**A**) shows typical sparse calcium transients of WT group. (**F**). Magnification of section highlighted in (**C**) shows example of burstiness observed in calcium traces of 5xFAD mice. (**G**) Histogram of inter-event intervals (IEI) for neuronal assemblies’ recordings, depicting enhanced count of short intervals for 5xFAD group, suggesting bursting activity (IEIs from all neurons, two-sample Kolmogorov–Smirnov = 0.0795, *p* = 4.47xE-140, neurons WT n = 1581, 5xFAD n = 1918, 5xFAD + Act n = 2515).

To investigate microcircuit network dynamics with single-cell resolution, we performed two-photon microscopy in layer II/III of the visual cortex in 5xFAD and WT mice (Fig. 2A). Upon bolus loading with the synthetic calcium indicator Oregon Green BAPTA-1AM (OGB-1;^45^), we labeled neuronal somata in WT (Fig. 2B; left panel) and 5xFAD animals (Fig. 2 B; right panel; note that cortical plaques would be clearly visible, as they are stained by OGB-1^14^). We recorded ongoing activity in the lightly anesthetized mouse^20,21^ and deconvolved the calcium transients (using *Online Active Set method to Infer Spikes* OASIS^46^, for details see SI Materials and Methods and Supplementary Fig. 1) for WT controls (Fig. 3A,B) and 5xFAD animals (Fig. 3C,D). We observed bursting patterns in a small fraction of neurons in the 5xFAD animals (Fig. 3C,F). Those bursts of activity were notably distinctive from the sparse appearance of calcium transients observed in the WT controls (Fig. 3E (WT), 3 F (5xFAD)). We quantified this phenomenon by analyzing Inter Event Intervals (IEI) of calcium transients, where bursting neurons should be represented by shorter intervals. Indeed, the IEI histogram showed an enhanced proportion of shorter IEIs in the histogram for 5xFAD as compared with WT controls (Fig. 3G, two-sample Kolmogorov–Smirnov = 0.0795, *p* = 4.47xE-140). We further analyzed the amplitudes of those calcium transients, and found lower amplitudes for 5xFAD mice than for WT controls (Chi-sq(2) = 563.32, *p* = 4.7589*E-123, mean ranks WT = 17,629, 5xFAD = 14,438, Supplementary Fig. 2).

When assessed at group level, we found that the IEI distribution in 5xFAD mice differed significantly from WT controls (Kruskal–Wallis H test, Chi-sq(2) = 57.48, *p* = 3.29xE-13; mean ranks WT = 84099.84, 5xFAD = 86016.23; Fig. 3G) with 5xFAD having, on average, higher mean IEI (5xFAD = 5564.11 ± 76.1 ms, WT = 3904.18 ± 36.4 ms). Interestingly, the average event rate also differed between groups, but with 5xFAD having lower event rate on average (5xFAD = 4.8 ± 0.26 events, WT = 10.6 ± 0.3 events; Kruskal–Wallis H test, Chi-sq(2) = 882.87, *p* = 1.93xE-192, mean ranks 5xFAD = 2899.26, WT = 4062.68). A useful visualization of IEIs belonging to individual cells’ transients is to plot their joint inter-event interval histograms, where the abscissa and ordinate represent the intervals preceding and following a transient^47^. Thus, in bursts, the initial transients are plotted at bottom right, the final transients are on top left, transients within bursts are at bottom left, and sporadic transients are at top right. For burst-dominated time series, most transients were indeed located near the mean interval value, on the lower left quadrant representing the enhanced proportion of transients occurring temporally close to each other (Supplementary Fig. 3, Table S1).

### Neuronal network dynamics de-organize and lose robustness in cortical microcircuits of 5xFAD mice

**Figure 4:**
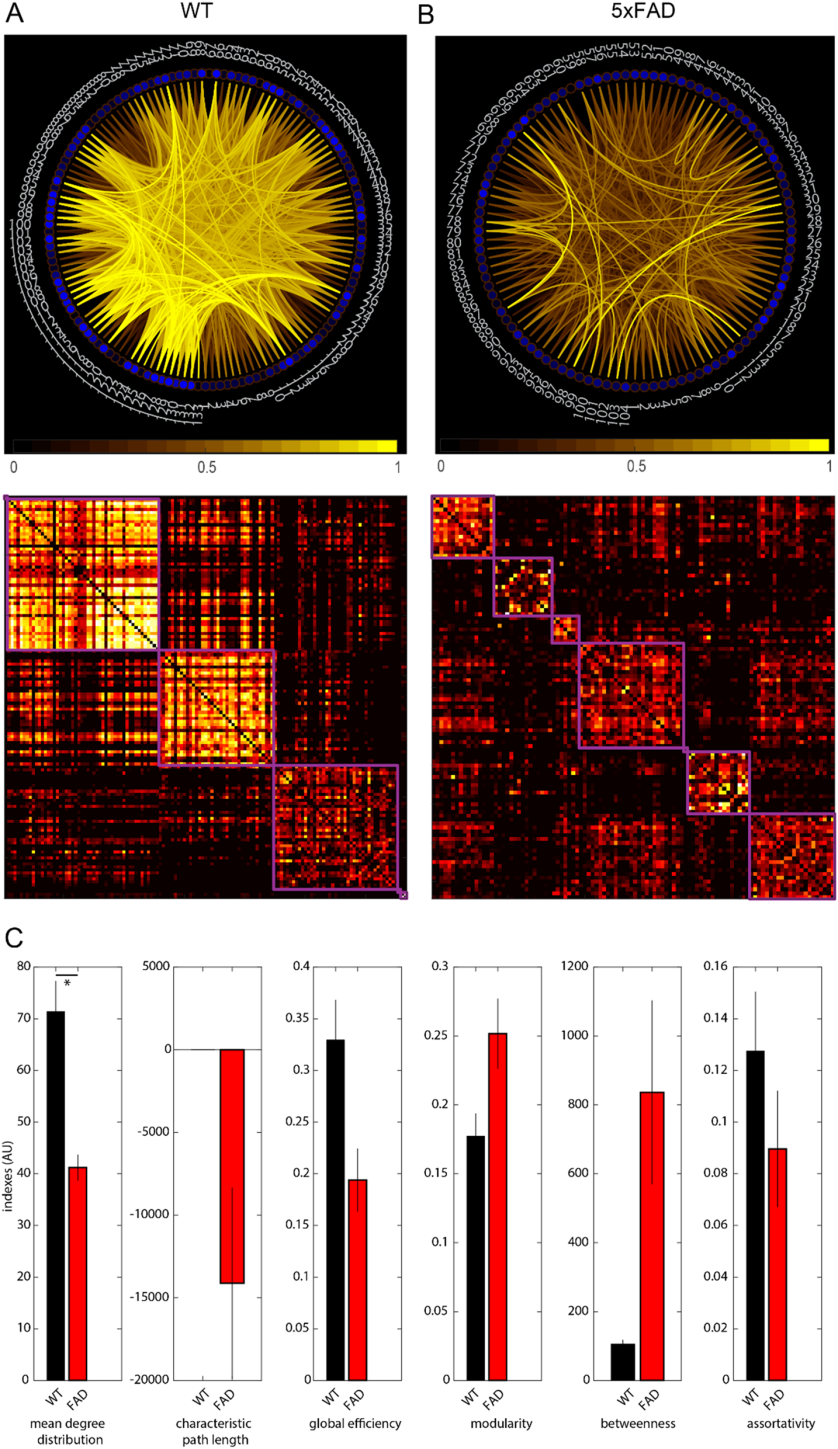
Graph theory-based measures of network dynamics reveal degradation of functional network topology in 5xFAD model. (**A**, **B**) Network internal connectivity (upper panels) and modular organization (lower panels) of representative networks differ in WT (**A**) and 5xFAD animals (**B**). Upper panels: Connections with higher than 50% of connection strength are depicted. Stronger hue indicates higher correlation coefficient. Numbered circles indicate network nodes (neurons’ somata). WT shows greater proportion of high correlation coefficients between nodes. Lower panels: Neuronal correlations of the same networks as left panels, sorted by modular organization with overlapping modules (purple). 5xFAD exhibits smaller modules as WT with lower correlations among its nodes. (**C**) Six complex network analysis indexes derived from graph theory for WT and 5xFAD groups (n = 5 mice each). For mean degree distribution, characteristic path length, global efficiency and assortativity, all measures of network robustness, the 5xFAD group shows lower values than the WT group. Each measure yields a value with internal scale in arbitrary units (AU). Reference values for biological networks are those observed in WT animals.

We next assessed network dynamics of cortical microcircuits with graph theoretic measures (Fig. 4A,B). Indeed, whereas visual capability of 5xFAD mice does not seem to be impaired at this stage (measured by visual discrimination test, data not shown), complex network measures of the imaged assemblies already differed between 5xFAD and WT.

Networks are described in terms of nodes and links^29^. We considered neuronal somata as network nodes, and their functional association (in the form of correlations) as network links. We assessed these functional profiles, derived from their calcium transients’ functional activity, in terms of different network parameters, which are relevant for robustness of cortical networks (^29^; Fig. 4C). We focused on *degree distribution* as an important marker of network development and resilience^29,48^ and thus a crucial differential marker between complex neural networks in health and disease. We observed a lower *mean degree distribution* in the 5xFAD group compared with WT control group (Fig. 4C; Kruskal–Wallis H test, Chi-sq(2) = 18.42, *p* = 0.0001, mean ranks 5xFAD = 17.22, WT = 39.45). We further assessed *global efficiency* and *characteristic path length* as a measure of integration, *modularity* as a segregation measure, and *assortativity coefficient*, as an additional index of network robustness^49^. On the latter indexes we found no or only marginal differences between groups (*characteristic path length* (Kruskal–Wallis H test, Chi-sq(2) = 0.31 , *p* = 0.85, mean ranks 5xFAD = 28.87, WT = 26.82); *global efficiency* (Chi-sq(2) = 4.89, *p* = 0.08, mean ranks 5xFAD = 22.35, WT = 34.27)*; modularity* (Chi-sq(2) = 3.55, *p* = 0.17, mean ranks 5xFAD = 32.09, WT = 22.64)*; betweenness* (Chi-sq(2) = 0.13, *p* = 0.93, mean ranks 5xFAD = 27.7, WT = 26)*; assortativity coefficient* (Chi-sq(2) = 1.76, *p* = 0.42, mean ranks 5xFAD = 24.91, WT = 32.55) (Tables S2, S3).

### Acitretin rescues network patterns and restores early behavioral deficits in 5xFAD mice

**Figure 5:**
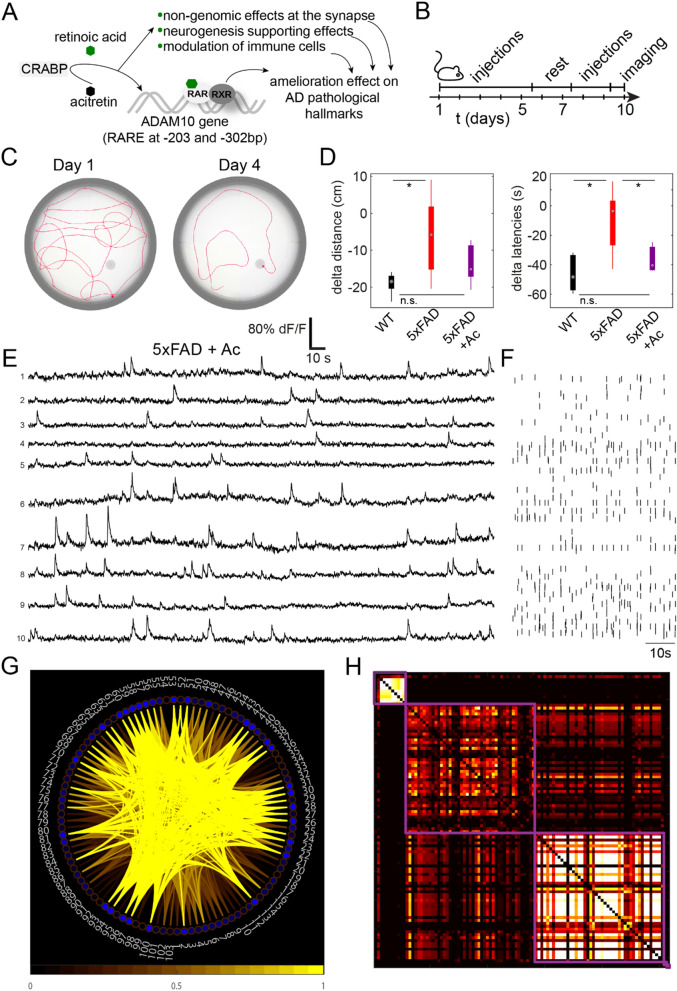
Acitretin treatment recovers network function both on single-cell level and on graph theoretical measures. (**A**) Proposed action-mechanism of acitretin. Acitretin itself was reported not to act as a direct ligand to retinoic acid receptors. However, it displaces retinoic acid from its cytoplasmic binding proteins (CRABP) and thereby increases intracellular availability of this bioactive ligand. Retinoic acid has been shown to exert different cellular mechanisms with potential benefit for AD. (**B**) Schematic of acitretin treatment (5 days of daily injection, 2 days of rest, 2 days of daily injections, imaging/behavioural testing). (**C**) Swim paths of representative animals of the 5xFAD group treated with acitretin, on day 1 and 4 of Morris water maze show improvement in finding the hidden platform after learning. (**D**) Differences between distance (left) and latencies to reach the hidden platform (right) for WT and 5xFAD animals in Morris water maze for WT, 5xFAD, and 5xFAD + Acitretin (Ac) groups. Treated 5xFAD mice do not differ significantly from WT animals (n = 5 animals per group). (**E**) Representative traces of 10 neurons from 5xFAD group treated with acitretin; ongoing activity in layer II/III neuronal assemblies in V1 cortex. (**F**) Raster plot from entire cell population from which selection in (**E**) was drawn shows calcium transient pattern across whole imaging session. (**G**) Network internal connectivity in the 5xFAD group treated with acitretin (n = 5 mice). Connections with higher than 50% of connection strength are depicted. (**H**) Modular organization of same network as in (**G**) in the acitretin-treated group. Neuronal correlations sorted by modular organization with overlapping modules (purple). Note that both correlation distributions and approximate size and number of modular organization resemble those observed in WT group (Fig. 4A).

Acitretin is a synthetic retinoid used in humans for treatment of psoriasis^50^. Re-purposing approaches for acitretin as a candidate AD drug revealed promising results in in vitro studies^38^. Its potential as an inducer of the non-amyloidogenic alpha-secretase ADAM10 has been subsequently demonstrated in primary cells, AD model mice, and a pilot study in human patients (^38,^^39,^^51^; Fig. 5A), indicating efficacy of the drug in mild to moderately affected patients^51^. A probable positive impact of acitretin during early stages of AD prior to plaque deposition and neuronal loss has not been investigated so far. We explored the effect of short-term acitretin treatment (Fig. 5B) on behavioral performance as well as on cortical microcircuit network dynamics. Acitretin treatment significantly improved behavioral impairments assessed by the water maze in 5xFAD mice, almost reaching wild type levels for *delta latency* (5xFAD + Ac = − 36.3 ± 3.9, WT = − 46.1 ± 5.6; t(8) = − 1.4115, *p* = 0.1958) and *delta traveled distance* (5xFAD + Ac = − 13.64 ± 2.4, WT = − 18.94 ± 1.4; t(8) = − 1.9030, *p* = 0.0935, Fig. 5C,D.). They also differed significantly from untreated 5xFAD mice in *delta latency* (5xFAD = − 10.5 ± 10.1, t(8) = 2.3796, *p* = 0.0446) albeit not in *delta traveled distance* (5xFAD = − 6.23 ± 5.1, t(8) = 1.3136, *p* = 0.2254).

Observation of the cortical activity pattern in acitretin-treated mice reveals regular and sparse patterns of calcium transients (Fig. 5E,F), resembling calcium transient patterns of WT controls (cp. Fig. 3A,B), and absence of the aberrant firing patterns observed in 5xFAD (Fig. 3C,F). Furthermore, the distributions of IEIs for the treatment group differed significantly from 5xFAD and were undistinguishable from WT controls (Kruskal–Wallis H test, Chi-sq(2) = 57.48, *p* = 3.2938xE-13, mean ranks 5xFAD + Ac = 83553.28, WT = 84099.84, 5xFAD = 86016.23). The calcium transients’ rate, however, was downregulated for the group treated with acitretin (Supplementary Fig. 3C).

Regarding network analysis measures, acitretin-treated 5xFAD mice diverged significantly from untreated 5xFAD animals, and showed values closer to WT controls, in the parameter *mean degree distribution,* critical for network resilience and development. For the networks recorded in the group treated with acitretin, *mean degree distribution* was significantly higher from the 5xFAD, and approached control values (Kruskal–Wallis H test, Chi-sq(2) = 18.42, *p* = 0.0001, mean ranks 5xFAD + Act = 32.75, WT = 39.45, 5xFAD = 17.22; Fig. 5G,H).

**Figure 6:**
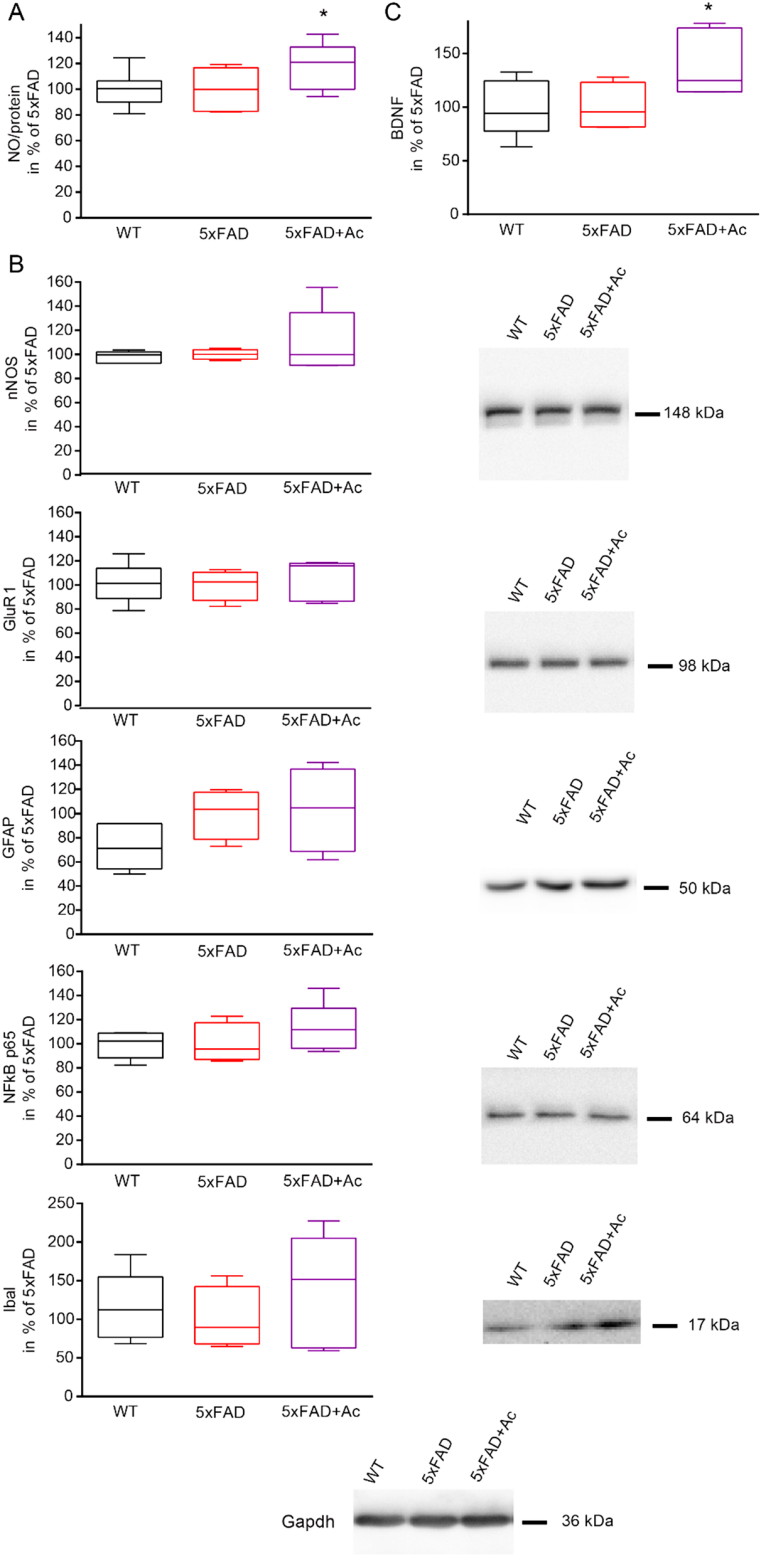
Impact of acitretin on neuronal network function and structure. (**A**) Quantitation of nitric oxide (NO) in visual cortex. (**B**) Expression of neuronal and glial markers in visual cortex following acitretin treatment. (**C**) Assessment of BDNF levels in brain homogenates. All measurements were done in groups of n = 4–5 animals. Treatment with acitretin was performed as described before. Data were analysed by Ordinary one-way ANOVA with uncorrected Fisher LSD (BDNF: *p* = 0.0496 for 5xFAD versus 5xFAD + Ac; NO: *p* = 0.029 for 5xFAD versus 5xFAD + Ac).

To decipher how acitretin might achieve stabilized network activity, we subsequently analyzed parameters indicating neuronal signaling, synaptic activity, and immune reaction within visual cortex (Fig. 6). Interestingly, nitric oxid production was elevated by acitretin administration (Fig. 6A) even if the synthesizing enzyme nNOS was not increased on protein level. At this comparably young age, no significant astrogliosis was detectable in the visual cortex, as indicated by GFAP quantification (Fig. 6B). Also IbaI and NFкb p65 levels were not altered; GluR1 as a representative of neurotransmitter receptors was also not affected. Lastly, we also assessed the amount of BDNF via ELISA and observed a significant increase in visual cortex due to acitretin administration (Fig. 6C). For full-length Western blots with corresponding marker bands see Supplementary Fig. 6.

### Excitatory synaptic density in 5xFAD is increased in layer II/III only, restored by acitretin treatment

**Figure 7:**
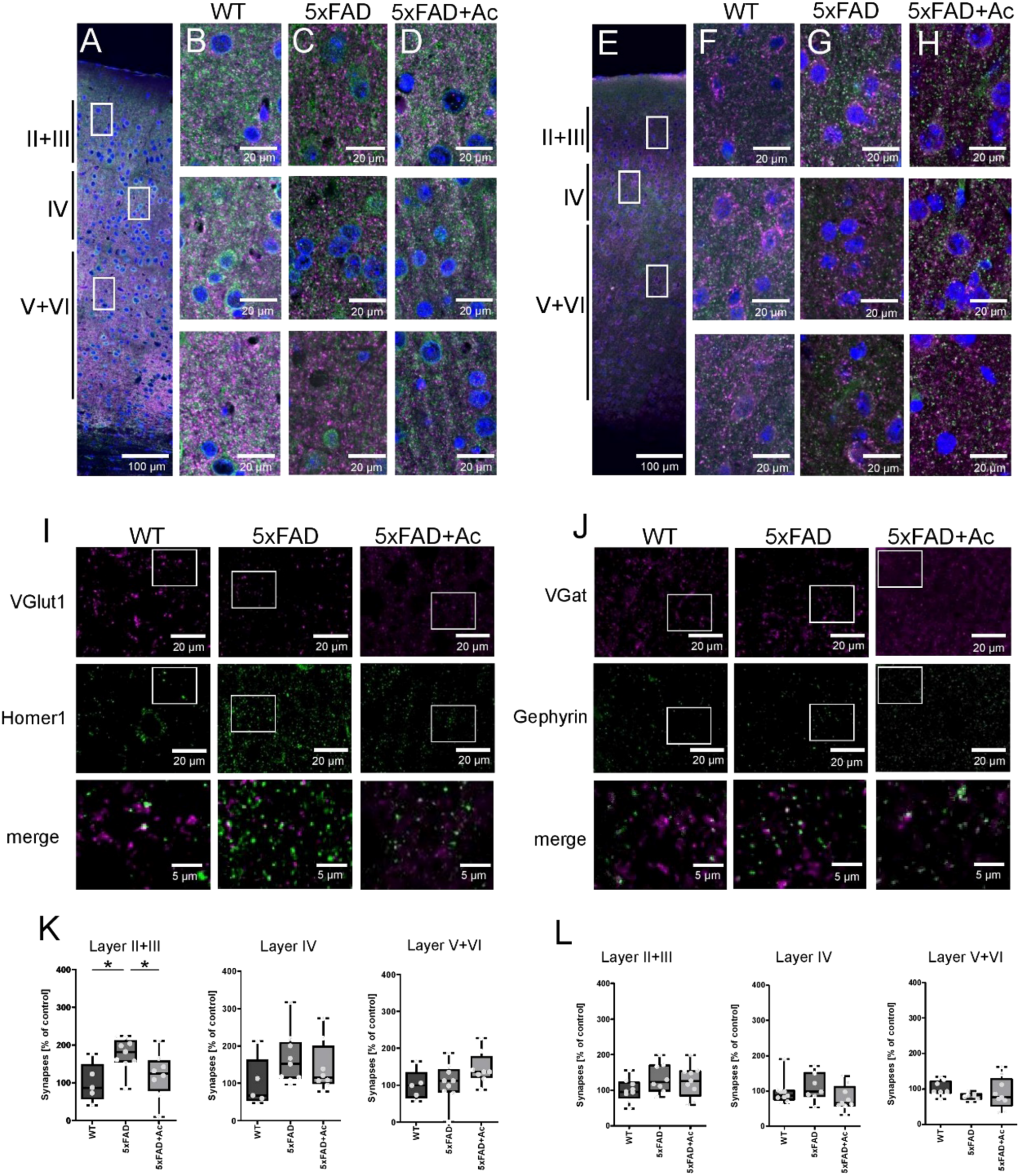
Number of excitatory synapses are increased in Layer II + III of 5xFAD mice. (**A**) Overview of V1 area in WT mice (green = Homer1, magenta = VGlut1, blue = DAPI). Rectangles indicate sites of examples pictures shown in (**B**). (**B**) Example pictures of Layer II + III, Layer IV and Layer V + VI of the V1 region in WT mice. (**C**,**D**) Example pictures of Layer II + III, Layer IV and Layer V + VI of 5xFAD and 5xFAD + Ac respectively. (**E**) Overview of V1 area in WT mice (green = Gephyrin, magenta = VGat, blue = DAPI). Rectangles indicate sites of examples pictures shown in (**F**). (**F**) Example pictures of Layer II + III, Layer IV and Layer V + VI of the V1 region in WT mice. (**G**,**H**) Example pictures of Layer II + III, Layer IV and Layer V + VI of 5xFAD and 5xFAD + Ac respectively. (**I**) Example pictures of pre- and postsynaptic clusters in WT, 5xFAD and 5xFAD + Ac mice. Shown is Layer II + III. The 3rd row shows merged images which represent excitatory synapses (Selections are indicated in the pictures above). (**J**) Example pictures of pre- and postsynaptic clusters in WT, 5xFAD and 5xFAD + Ac mice. Shown is Layer II + III. The 3rd row shows merged images which represent inhibitory synapses (Selections are indicated in the pictures above). (**K**) Excitatory synapse counts in Layer II + III, Layer IV and Layer V + VI. Quantification shows that excitatory synapse numbers are selectively increased in Layer II + III of 5xFAD mice in comparison to WT or 5xFAD + Ac. (**L**) Inhibitory synapse counts in Layer II + III, Layer IV and Layer V + VI. Quantification shows that inhibitory synapse numbers are not changed. Box plots indicate whiskers of the 5–95 percentile. Dots represent single data points. *P* values were determined using one-way ANOVA followed by Dunnett’s multiple comparisons test. Data are from 3 animals with 3 slices each.

The altered firing patterns could be underpinned by changes in synaptic densities, and said changes may, or not, be layer specific. To contrast this hypothesis, we conducted immunohistochemical stainings of V1 coronal sections with antibodies for all experimental groups (Fig. 7A-H), demarcating excitatory (anti-VGlut1, anti-Homer1) and inhibitory synapses (anti-VGat, anti-Gephyrin). Importantly, we found a selective increase in the density of excitatory synapses (Fig. 7I,K) in layer II/III only. Excitatory synapses in other layers (Fig. 7K, Supplementary Fig. 5) and inhibitory synapses in all layers analyzed were not affected, neither by the genotype, nor by acitretin treatment (Fig. 7J,L). Moreover, the increase in excitatory synapse levels in layer II/III was completely restored upon acitretin treatment. This finding suggests, that indeed, the dysregulation of the functional network in layer II/III is accompanied by changes in excitatory synapse numbers. Notably, layer II/III integrating intracortical connections appears to be considerably plastic in this context, compared to the other cortical layers.

## Discussion

For microcircuit data, graph theoretical approaches have not been applied, mostly due to restrictions on the generalization of certain network parameters across individuals. Arguably, some measures of local integration have different interpretations at microcircuit level, as for whole brain analysis to which graph theory has classically been applied. However, it is possible to characterize network parameters for each local network imaged, and subsequently compare indexes among groups in order to establish differences in network integration. In this study, we explored ongoing neuronal interaction dynamics in layer II/III of V1 in a mouse model resembling amyloid pathology of familial AD, both on the single-cell and the topological level. Since we investigated animals prior to the onset of pronounced cortical plaque deposition, neuronal network qualities should not reveal changes due to mere loss of neurons but instead due to early functional changes. We therefore applied graph theory to probe network vulnerability and impairment in an early stage of AD.

### Complex network measures capture early functional disruption in AD

In human AD patients, apparent cognitive deterioration begins when a large number of neurons has already been lost^52^, leaving clinicians little time to attempt treatment. A plausible explanation for this notably initial robustness of the system to sustained neuronal loss is the capability of the neuronal network to supply the function of lost neurons by distribution among other nodes, i.e., unaffected neuronal ensembles present in the network^29^. Following this rationale, any behavioural impairment must be preceded by subtle network disruptions which, if detected, could carry diagnostic value. Thus, it is relevant to characterize network dynamics in search of signs of early disruptions, explore the networks’ robustness in regards to vulnerability to damaged nodes, and further assess whether they correlate with pre-clinical symptoms. In our study, we found only one of the network indexes to be altered in 5xFAD mice V1. This suggests that at this early stage, only subtle changes of neuronal communication modes become apparent, in accordance with the finding that loss of pyramidal neurons in 5XFAD models does not commence until 9–12 months of age^44,53^. It is plausible that *mean degree distribution* would have been more sensitive than other markers (i.e. assortativity coefficient). Considering that distributions of values can constitute a more global description of the network, with their central measurements acting as a more stable marker of a network feature, in this case, network resilience^29,54^.

Few neurons exhibited bursting calcium-transient patterns in the AD model, captured by their IEI distributions. The minimal proportion of cells with burst-like aberrant activity might play a key role in the distortion of neuronal communication, possibly by enhancing noise beyond functional levels, thereby impacting higher level network dynamics. Graph theory predicts scale-free topologies including hubs (i.e., highly interconnected neurons) to be an effective design to organize network communication^55^. With our approach we can only image the present state of the recorded neurons’ functional network as their connection degrees are defined dynamically as correlation. The current state of a neuron would not inform us about its past functional role, given that the correlation with other neurons eventually has changed and a neuron that was a highly linked hub neuron before neurodegeneration, might cease to be a hub after functional collapse. Aberrant firing patterns have more detrimental effects on network communication if the abnormal cell is a hub neuron with a high number of connections (degree). Arguably, if a fraction of those neurons would exhibit the aberrant patterns we observed in our data, their impact on network dynamics would be amplified by their central role in network communication. Thus, even a small proportion of aberrant firing patterns would ultimately lead to the functional deterioration observed in neurodegenerative diseases. Further *in-silico* work might shed light on this hypothesis, for example setting up a small network including model neurons with normal activity parameters and exploring the potential disruptions elicited by the introduction of a small population of neurons mimicking the aberrant activity patterns reported here. This could shed light on the time required for a small population with aberrant firing patterns to induce a noticeable disruption at global network level.

### Relevance of ongoing activity to study neuronal network dynamics

Keeping neuronal ensembles in a constant and stable state is a fundamental condition for the interpretation of network analysis parameters to be meaningful. We chose the lightly sedated state to reduce physiological noise and keep the network’s intrinsic state constant as during awake periods neural networks undergo constant state fluctuations^56–58^. These changes occur randomly during wakefulness as patterns of activity vary on slow and rapid time scales, shaping the ongoing signal^58,59^. Such moment-to-moment fluctuations during waking^58,60^ would not necessarily influence the aberrant firing of neurons, but the rest of the network, adding multi-dimensional activity^56^. As recently, findings of early hyperactivity in a mouse model of Huntington´s disease in this controlled state^20^ have been recapitulated in awake animals^61^, we opted for the lightly sedated condition as the basis for graph theoretical measures.

### Acitretin reverses network measures, protein expression and synapse densities in 5xFAD mice

Subchronic treatment of 5xFAD mice with acitretin stabilized abnormal network activity with regard to firing patterns and distributions of mean IEI, and reversed behavioural performances in MWM. Aβ increases spontaneous neuronal activity early during disease progression in the absence of amyloid plaques or neurofibrillary tangles^62^. We previously reported that single stereotactic application of acitretin in another AD mouse model (APP/PS1) was sufficient to re-balance APP processing with a strong reduction of Aβ^38^. By activating ADAM10 transcriptional activity, the alpha-secretase that prevents Aβ synthesis^63^, acitretin is able to lower A-beta production^43^. The amount of soluble Aβ was comparably low in the cortex of 5xFAD mice at the investigated time point. Small reductions in Aβ synthesis might still be beneficial in an early state, while in a pronounced pathological state, efficacy of ADAM10-enhancement by such subtle changes might be not sufficient to have beneficial effects. Another possible mechanism for the reversal effects in the treated 5xFAD animals might be due to elevation of IL-1^64^ or IL-6^65^ evoked by acitretin. The activation of a cytokine network in the brain might play an important role in plasticity and can be relevant for LTP maintenance, for example an overexpression of IL-6 might be able to trigger the outgrowth of new fibers^66^.

Acitretin itself is not a ligand to retinoic acid receptors. It has been shown to replace retinoic acid from intracellular binding proteins and thereby increase the available pool of this bioactive compound^67^. Several studies found an increase of neuroprotective gene expression by retinoic acid receptors: e.g., elevated expression of neurotrophic tyrosine kinases 1 and 2 in human neuroblastoma cells^43^. Administration of β-carotene, the precursor of retinoic acid, in an autism mouse model increased BDNF concentration^68^. More recently, homeostatic synaptic plasticity at inhibitory synapses in murine visual cortical circuits has been shown to depend on retinoic acid signaling indicating a potential inhibitory mode of action^69^. We did not observe increased amounts of GluR1 in the cortex. However, nitric oxide levels were increased by acitretin treatment. NO/cGMP signaling participates in synaptic plasticity in the visual cortex^70^ and. another synthetic retinoid receptor ligand – AM-80 – linked retinoid signaling via NO to ERK-dependent BDNF synthesis in midbrain dopaminergic neurons^71^. We observed an increase of this neurotrophic factor in visual cortex tissue. BDNF suppresses responses to exogenously added glutamate in slices from rat visual cortex^72^ and promotes development of dendrites and synapses of cultivated rodent V1 GABAergic neurons^73^. These factors, taken together might explain the restoration of homeostatic network conditions we observed for acitretin-treated animals.

Notably, acitretin was capable of rebalancing the increased density of excitatory synapses specifically in layer II/III of visual cortex in 5xFAD mice. Previous work suggested maladaptive synaptic plasticity changes to be related to network dysregulations in mouse models of relapse-remitting MS^20^. While the pathophysiological mechanisms of relapse-remitting MS and early stage AD are distinct, it is plausible that the early dysregulations in cortical regions we observed, represent a plasticity driven phenomenon. This notion is supported by the fact that acitretin treatment restored excitatory synapse counts, alongside with rebalancing network parameters and ameliorating behavior. Hence, acitretin seems to be a valuable therapeutic approach in the early stage of AD.

### Network-based analyses of cortical microcircuits indicate treatment-related functional restoration in early-stage neurodegenerative disorders

Increasing evidence points to the particular importance of studying cortical network dynamics in early stages of neurodegenerative and neuroimmunological disorders as diverse as Huntington’s disease^20^, AD^18^, and relapse-remitting MS^21^. Importantly, the first studies of cortical neuronal assemblies in a mouse model of AD described hyperactivity in the late stage of the disease, dependent on the spatial proximity to plaques^12^. These individual hyperactive neurons most likely change their activity state due to the cytotoxic environment. Here, we describe a distinctively different, early, type of network dysregulation, which might be closely related to (mal-) adaptive changes in the network. Indeed, the selective increase in excitatory synapse densities in layer II/II which could be rebalanced by acitretin treatment points to these early, subtle changes to be of different origin than the later changes. While the cortex is not the first brain region affected by the molecular pathology in neither of these disorders, it seems to react in a maladaptive fashion prior to the onset of neuronal loss and apparent protein aggregates or T-cell infiltrations. Our choice to image neuronal ensembles in V1 was driven by the evidence reported by other groups showing that visual cortex represents an area which is compromised in AD progression directly after medial entorhinal cortex, so relatively early on during the disease^74,75^. These early-stage cortical network dysregulations might indeed represent a primary target of preventive therapies. Our study suggests that using computational methods dedicated to the analysis of cortical network interactions, in combination with the application of therapeutic targets, might provide new ways of understanding the local cortical network as a pathophysiological entity.

Network analyses as used here, rely on information related to response amplitude of calcium transients, which are highly non-linear in GCaMP-based measurements. For synthetic calcium indicators as OGB-1 fluorescence changes from successive action potentials combine nearly linearly^76,77^ and can be inferred by deconvolution (e.g.^78,79^). The same inferences remain challenging for imaging data based on genetically encoded calcium indicators as the associated responses are complex, nonlinear and variable over neurons^80–82^. This nonlinearity and variability can be explained by GCaMP’s four calmodulin-derived binding sites, dependence of fluorescence amplitude and shape of transients on total GCaMP concentration depending on expression, as well as by its lower dynamic range compared to OGB-1^81^. The neuronal networks in our case are functional, as opposed to structural networks. In a functional network, the edges or connections between the (neuronal) nodes are defined by a measure of functional association, commonly the Pearson’s product-moment correlation coefficient, which captures the strength and direction of a monotonical relationship between two variables, in this case neuronal activity levels.

Graph theory is commonly used for gathering information about interactions of whole-brain networks by treating brain regions as nodes, and highly interconnected areas as hubs. Employing the same analytic approach on the microcircuit level allows to monitor the influence of disturbances in the temporal distribution of single-cell activity on the topology of the local network. In contrast to studies in late stage AD^12^, we did not find a “hyperactivity phenotype” close to plaques, but a rather complex dysregulation of local neuronal networks, potentially underpinned by synaptic density. In contrast to human brain network modelling, a node in the local neuronal network index the activity of an individual neuron. Abnormal activity of individual cells eventually leads to a breakdown of the higher-order interactions in the local network which might serve as the seed of brain-wide functional degradation. Further investigation could shed light on the “tipping-point” where local networks break down of too many components exhibit aberrant activity. A neurodegenerative disease is a complex collection of events at multiple levels. Combining graph theoretical analysis, promising drugs such as acitretin, and synaptic density counting, holds diagnostic and therapeutic value. Early intervention yields the opportunity to restore cortical network dynamics while the disease is still in its prodromal stage, thereby granting valuable time to attempt further treatment.

## Supplementary Information

Supplementary Information
