# Supplementary material for: Acitretin reverses early functional network degradation in a mouse model of familial Alzheimer’s disease
Source: Sci Rep. 2021 Mar 23;11:6649. doi: 10.1038/s41598-021-85912-0 (PMC7988040; doi:10.1038/s41598-021-85912-0)
Supplement: Supplementary file 1 — Supplementary Information [file 41598_2021_85912_MOESM1_ESM.docx]

**Acitretin reverses early functional network degradation in a mouse model of familial Alzheimer’s disease**

Eduardo Rosales Jubal^1,2,3^, Miriam Schwalm^1,2,4,5^, Malena dos Santos Guilherme^6^, Florian Schuck^6^, Sven Reinhardt^6^, Amanda Tose^1,2,7^, Zeke Barger^1,2,7^, Mona K. Roesler^8^, Nicolas Ruffini^9^, Anna Wierczeiko^9^ Michael J. Schmeisser^1,8^, Ulrich Schmitt^6^*, Kristina Endres^6^* & Albrecht Stroh^1,2,9^*

^1^Focus Program Translational Neurosciences, University Medical Center of the Johannes Gutenberg-University Mainz, Germany

^2^Institute for Pathophysiology, University Medical Center of the Johannes Gutenberg University Mainz, Germany

^3^Competence Center for Methodology and Statistics, Luxembourg Institute of Health, Strassen, Luxembourg.

^4^GRADE Brain, Goethe Graduate Academy, Goethe University Frankfurt am Main, Frankfurt am Main, Germany

^5^Department of Biological Engineering, Massachusetts Institute of Technology, Cambridge, MA, USA.

^6^Department for Psychiatry and Psychotherapy, University Medical Center of the Johannes Gutenberg-University Mainz, Germany

^7^Department of Molecular and Cell Biology, Helen Wills Neuroscience Institute, UC Berkeley, CA, US

^8^Institute for Microscopic Anatomy and Neurobiology, University Medical Center of the Johannes Gutenberg-University Mainz, Germany

^9^Leibniz Institute for Resilience Research, Mainz, Germany

* equally contributing last authors

Correspondence:

Prof. Dr. Albrecht Stroh

Institute for Pathophysiology

Johannes Gutenberg-University Mainz

Hanns-Dieter-Hüsch-Weg 19, 55128 Mainz, Germany

Email: albrecht.stroh@unimedizin-mainz.de

Phone: +49 6131 39-21347

Dr. Eduardo Rosales Jubal

Competence Center for Methodology and Statistics

Luxembourg Institute of Health

1A-B, rue Thomas Edison, 1445 Strassen, Luxembourg.

Email: eduardo.rosalesjubal@lih.lu

Phone: +352 26970-954

**Supplementary material**

Supplementary text: **SI Materials and Methods**

**Figures S1 to S4**

**Tables S1 to S4**

**SI References**

Supplementary Information Text

SI Materials and Methods

Animals

Male 5xFAD mice (Jackson Laboratory, Bar Harbor, ME, USA) were crossbred for maintenance with female C57BL/6J from the animal facility of the University Medical Center of Mainz. Food and water were provided ad libitum and a 12 h light–dark cycle was maintained. Genotyping was performed after weaning as described before ^1^. Animal husbandry and experimental manipulation were carried out according to animal welfare guidelines of the Johannes Gutenberg-University Mainz and were approved by the Landesuntersuchungsamt Rheinland-Pfalz, Koblenz, Germany. All animals used in this study were 16 week old males. Age-matched wild type littermates were used as controls.

Two-photon imaging

Craniotomy preparation and dye injection. Mice were anesthetized with Isoflurane (Forene, Abbott, Wiesbaden, Germany) mixed with pure oxygen to maintain surgical depth of anesthesia (3.0 % induction, 1.5–2.0 % maintenance). Animals were fixed on a stereotactic frame with ear- and bite bars and placed onto a warming plate to keep body temperature constant at 37°C throughout the surgery. Anesthesia depth was repeatedly assessed by monitoring tail-pinch reflex and respiration rate. After application of topical anesthesia (Xylocain Gel 2%, AstraZeneca, Wedel, Germany) a skin incision was made and the exposed area of the skull was cleaned and dried from fluids and blood. Stereotactic coordinates of binocular visual cortex (V1) were located accordingly (anteroposterior (AP) -3.8 mm, mediolateral (ML) 2.5 mm; ^2^) and a craniotomy of approximately 1x1 mm was prepared under a dissection microscope (Leica, Wetzlar, Germany) using a dental drill (Ultimate XL-F, NSK, Trier Germany, and VS1/4HP/005, Meisinger, Neuss, Germany) and a scalpel or a needle to remove the skull bone. The exposed area was moistened with 37°C warm Phosphate Buffered Saline (PBS, Sigma, Munich, Germany). Above the craniotomy a custom-made metal chamber was glued onto the skull using cyanoacrylic glue (UHU GmbH, Buhl-Baden, Germany) and dental cement (Heraeus Kulzer GmbH, Hanau, Germany). After a waiting period allowing the glue and cement to dry, the animal was transferred onto the heating plate of the two-photon microscope while being supplied with 0.6-0.8% Isoflurane. The metal chamber was continuously perfused with warm (37°C) artificial cerebrospinal fluid (125 mM NaCl, 2.5 mM KCl, 1.25 mM NaH2PO4, 26 mM NaHCO3, 2 mM CaCl2, 1 mM MgCl2, 20 mM Glucose, pH 7.4) or PBS. The fluorescent calcium indicator Oregon Green Bapta-1 AM (OGB-1; 500 μM; Life Technologies/ Molecular Probes, Waltham, USA) was bulk loaded into the cortex. Briefly, the indicator dye was dissolved in 20% pluronic F-127 dimethyl sulfoxide (DMSO; Thermofisher Life Technologies, Darmstadt, Germany) and the solution was further diluted with standard pipette solution to obtain a final concentration of 0.5 mM. The diluted and filtered dye was back-loaded into glass pipettes of 4-6 MΩ resistance (Sutter Instrument Inc., Novato, USA) and pressure-injected (0.6 - 0.8 bar for 4 min) around 200–300 μm below the intact dura using a picospritzer (Scientifica Pvt Ltd., Berkshire, UK) as described previously ^3^. 30–60 min after dye-loading, imaging was performed. To reduce movement artifacts caused by breathing or heartbeat of the animal, the craniotomy was filled with 0.8 – 1% agarose (Sigma Aldrich, Missouri, USA) in some of the recordings.

Image acquisition. For in vivo imaging, a custom-built two-photon microscope based on a Ti:Sapphire laser (Chameleon; Coherent systems, California, USA) , e.g Ti:Sapphire (Ti:Sa) with 700 – 1000 nm tunable output, equipped with a resonance scanner, operating at 800 nm wavelength (LaVision Biotec, Bielefeld, Germany) was used. The intensity of the laser was modulated with a Pockel’s cell. The scanning was performed using a 25x (1.1 N.A.; model-nr: MRD77220. Nikon, Tokyo, Japan) water immersion objective mounted on an upright microscope. The emitted fluorescence was detected using a photomultiplier tube (PMT, Hamamatsu Photonics, Hamamatsu, Japan). All acquired images were collected full-frame, either at 15 or at 30 Hz using custom-written software (Imspector Pro; LaVision Biotec). At each focal plane, spontaneous calcium transients of layer 2/3 neurons were recorded for at least 6 min for each trial (5.400 - 10.0000 images in one timelapse). For simultaneous visualization during scanning, the emitted fluorescence of OGB-1 labeled neurons was collected at 515 nm (515±20 nm filter).

Image analysis. Data analysis was performed off-line using custom-written MATLAB scripts (Mathworks, Nathick, MA, USA). Neurons were visualized by averaging pixels across image sequences and regions of interest (ROIs) were semi-automatically drawn around individual neuronal somata by tracing the outlines of cell bodies on a single image. ROIs were selected after assessment of the average intensity or standard deviation z-projection of the image stack, combined with careful visual examination of individual imaging frames. Absolute ROI fluorescence was defined as the averaging all pixels and dF/F values were obtained after referencing the absolute values to a baseline corresponding to a silent period in each ROI. Thereafter the result was rasterized by converting peak as 1 and the rest, including baseline, as 0. Astrocytes were excluded from analysis upon identifying their specific morphology and typical transients ^4-7^.

Deconvolution of calcium traces by Online Active Set method to Infer Spikes (OASIS). The OASIS method was developed by Friedrich and colleagues^8^ from a generalization of the Pool Adjacent Violators Algorithm (PAVA) for isotonic regression. In this approach, the calcium transient is modelled as an autoregressive process of order p (AR(p)) where p is a small positive integer, usually 1 or 2. For details on the algorithm, please refer to their original publication ^8^. Briefly, the OASIS algorithm progresses through the fluorescence time series sequentially from beginning to end, verifying violation in constraints. A constraint violation encountered for a time step leads to backtracking and merging. The algorithm proceeds moving forward until the next violation occurs and triggers backtracking and merging as long as constraints are violated. When the most recent spike time has been reached, the algorithm proceeds forward again. The process continues until the end of the series has been reached (Friedrich et al, Fig. 2). When a solution is obtained the pools correspond to the Inter Spike Intervals (ISI). Here we used the provided MATLAB implementation (https://github.com/j-friedrich/OASIS), with AR(1) model for calcium dynamics.

Network Analysis

We explored neuronal networks following the steps defined in ^9^:

*- Define network nodes*: in our case, the nodes were neuronal somata, as the biological unit susceptible to explain variance on the investigated phenomena and current technique.

*- Estimate a continuous measure of association between nodes:* this constituted the edges or links of the network. In our case, we used the correlation between nodes calculated with the Pearson product-moment correlation coefficient. Correlation matrices were calculated on the deconvolved traces generated by the OASIS algorithm.

*- Generate an association matrix compiling all pairwise correlations:* the matrices containing the correlation coefficients between the fluorescent signals of OGB-1 positive neurons in the field of view were thresholded (p=0.5) to create adjacency matrices.

*- Calculate the network parameters of interest and compare them among experimental groups.* The thresholded adjacency matrices from the previous step represented weighted undirected networks were the input for further network analysis.

Considering the nature of the association measure between their nodes, our dataset consisted of functional networks. Thus, only complex network measures which can be applied and interpreted in functional networks were assessed, as some complex network measures are only meaningful when derived for structural networks (i.e. where links are anatomical connections) (32). The network measures were derived from the adaptation of the Complex Networks Analysis toolbox The Brain Connectivity Toolbox for MATLAB (34). Per experimental group 5 animals were imaged and these data were used for the network analysis described.

**Network measures**

**Mean degree distribution:** The degree of a node is the number of edges that connects it to the rest of the network. Degree is the most fundamental measure in a network and many other measures are derived or linked to node degree ^9^. The collection of the degrees of all nodes in a network form a degree distribution ^10^, commonly referred as an important marker of network development and resilience. The mean network degree is frequently used as a measure of density, or the total “wiring cost” of the network ^11^.

**Characteristic path length:** is the average shortest path length between all pairs of nodes in the network. A path length is the minimum number of edges that must be traversed to go from one node to another. This is the most commonly used measure of functional integration ^11^.

**Global efficiency:** the average inverse shortest path length, deemed more suitable to measure network integration ^12^, given that it is primarily influenced by short paths and may be meaningfully computed on disconnected networks. For paths between disconnected nodes are defined to have infinite length, and thus zero efficiency. Functional networks tend to be highly segregated but have a lower global efficiency.

**Modularity:** a module consists of multiple densely interconnected nodes, and it constituents nodes are comparatively less connected with other modules’ nodes. complex networks consist of a number of modules ^9^. A variety of algorithms exists to estimate modularity in a network, many of them based on hierarchical clustering ^13^.

**Assortativity coefficient:** the assortativity coefficient captures the correlation between the degrees of connected nodes. A positive assortativity coefficient indicates that nodes tend to functionally connect to other nodes with the same or similar degree ^9^. Networks with a positive assortativity coefficient might share a core of interconnected high-degree hubs likely yielding them a comparatively higher resilient ^11^.

**Betweenness:** fraction of the shortest paths that pass through a given node. Bridging nodes that connect disparate parts of the network tend to have a high betweenness centrality ^11^.

A comprehensive list of network measures are available at Brain Connectivity Toolbox website <https://sites.google.com/site/bctnet/measures/list>.

Quantification and Statistical analysis

We determined the sample size based on previous publications reporting in-vivo calcium imaging in awake and lightly anesthetized mice. Statistical measures were calculated in the parametric space, when attributes of data distributions, normality (Lilliefors test) and homoscedasticity criteria allowed it, or with non-parametric statistics otherwise. Since the three experimental groups were collected at the same time, we selected statistical tests for more than two groups. The initial exploration was using the Kruskal Wallis test, a non-parametric alternative to a one-way ANOVA, followed by post-hoc multiple comparison analysis to establish further differences between groups with the Tukey-Kramer method. The analysis of the two photon microscopy data, correlations, and network measures was performed in MATLAB (Mathworks. Natick, MA). Statistics were calculated either with the MATLAB Statistical Toolbox package, GraphPad Prism 6 (Graph Pad Software, La Jolla, CA), or SPSS (IBM, Ehningen, Germany). Values are reported as mean ± SEM for normally distributed data. When non-parametric statistics were used, mean ranks are reported (see also Supp. Table 1 for a summary of descriptive statistics for graph theoretical parameters).

Behavioral testing

Morris Water Maze. Spatial learning and memory was tested by the Morris water maze hidden platform task using the same maze and protocol as described ^14^. In brief, the platform stayed in the same quadrant for all trials and the animals were released from four different positions at the pool perimeter. Mice performed four trials per day on four consecutive days with a maximum length of 60 s and an inter-trial interval of 90 s. Mice were allowed to stay on the platform for 10 s. Learning was assessed by measuring the latency to reach the platform and the distance to the platform, expressed as difference from day 1 to day 4.

Monitoring of behavior. For Morris water maze a computerized video system registered moving-path and duration automatically. The hardware consisted of an IBM-type AT computer combined with a video digitizer and a CCD video camera. The software used for data acquisition and analysis was EthoVision XT® release 8.0 (Noldus Information Technology, Utrecht, Netherlands).

Acitretin treatment

5xFAD Mice were given a 9 days treatment, consisting of 5 consecutive days of one daily injection, 2 days of rest, 2 days of daily injections. Acitretin solution was prepared freshly short before the injection by dissolving 1 mg acitretin per ml corn oil and kept light-protected during manipulation. Mice received a dosage of 10mg/kg in a total volume of 400 µl corn oil via i.p. injection. On day 10 the animals were tested either behaviorally or in two photon calcium imaging (Figure 5 B).

Dissection and histology

Brains were dissected, washed with 0.9% NaCl and immediately submerged in 4.5% formalin for 24h at RT. Subsequently, hemispheres were embedded in paraffin and sections were stained using the primary antibody 6E10 (described before in ^15^). Microscopic pictures were taken with 10x10 magnifications (EVOS XL, Life Technologies, Darmstadt, Germany; equipped with L Plan PH2 10x/ NA 0.25 objective).

Immunohistochemistry

Animals (16 weeks) were transcardially perfused with 1x PBS. Brains were post-fixed in 4% PFA in PBS before preparing 100 µm vibratome sections. For each genotype/condition 3 slices of 3 animals were analyzed. An additional slice was used as a control and only treated with secondary antibodies. Layers II/III, layer IV and layers V/VI were detected in each slice using DAPI staining. Slices were washed 3 times for 5 min in 1x PBS before they were incubated in blocking solution (20% BSA, 0.2% Triton in 1x PBS) for 2h at RT. The solution was exchanged with fresh blocking solution (10% BSA, = 0.2% Triton in 1x PBS) including primary antibodies for 48h. The following antibodies and concentrations were used: DAPI (D1306 Thermo-Fisher Scientific, 1:1000), anti-VGlut1 (chicken, 135316, Synaptic Systems 1:250), anti-Homer1 (rabbit, 160023, Synaptic Systems, 1:250), anti-VGat (guinea pig, 131004, Synaptic Systems, 1:250), anti-Gephyrin (rabbit, 147018, Synaptic Systems, 1:250). Slices were again washed 3 times for 5 min each in 1x PBS before secondary antibodies were added in fresh blocking solution for 2h at RT. The respective secondary antibodies were used Alexa647-conjugated anti-mouse (goat, A-21235, Thermo-Fisher Scientific; 1:1000), Alexa568-conjugated anti-chicken (goat, A-11041, Thermo-Fisher Scientific; 1:500), Alexa488-conjugated anti-rabbit (donkey, A-21206, Thermo-Fisher Scientific; 1:1000) Alexa568-conjugated anti-guinea pig (goat, A-11075, Thermo-Fisher Scientific, 1:500). DAPI was added into the same solution for 15 min at RT before the slices were washed 3 times in 1x PBS for 5 min each. The slices were then mounted with ProLong Gold Antifade (P36930, Thermo-Fisher Scientific) and imaged with a confocal microscope (Leica TCS SP8, 63x objective).

Analysis and statistics. Images were taken from V1 cortical layers. To determine the number of pre- and postsynaptic clusters and the number of synapses, the synapse counter plugin of Fiji ^16^ was used as in ^17^ with the following parameters: rolling ball radius 1, maximum filter radius 2, threshold adjustment Otsu, pre- and postsynaptic particle size 12 voxels, maximum pre- and postsynaptic particle size 750 voxels. Analyses were performed using GraphPad Prism 5 (GraphPad Software, Inc.). All data sets were subjected to Shapiro-Wilk normality test. For comparison of more than two normally distributed groups, the presence of significant differences was first evaluated using ordinary one-way ANOVA, followed by Dunnett’s multiple comparisons test against the control values. For comparison of more than two not normally distributed groups, the presence of significant differences was first evaluated using ordinary Kruskal-Wallis test, followed by Dunn’s multiple comparisons test against the control values.

Aβ and BDNF ELISA

To quantify the amount of soluble and insoluble plaque-associated Aβ42 peptides in the cortex, we used a two-step protocol with diethylamine- (soluble) and formic-acid-extraction (insoluble) according to protocols published previously (e.g. ^18^). Mice (4 months, n=4 per group) were anesthetized by isoflurane and sacrificed. Right hemispheres of mice were used for dissection of cortex using a pre-chilled metal surface for preservation of tissue integrity. Tissue was harvested and soluble material as well as formic acid extracted material used for measurement of human Aβ42 peptides. Tissue was snap-frozen in liquid nitrogen and stored at -80°C until further use. Subsequently, tissue samples were homogenized using 0.2% DEA solution at a concentration of 50 or 400 mg tissue/mL in a tissue mill with stainless steel beads (2 min 20 Hz, Qiagen). Homogenates were centrifuged at 100,000 x g for 1 h at 4°C and aliquots of supernatant (containing soluble Aβ) neutralized by adding 1/10 volume 0.5 M Tris HCl pH 6.8. The residual material was substituted with 70% formic acid (200 µl for cortex) and each sample sonicated individually for 1 min on ice. Insoluble material was spun down at 135,000 x g for 1 h at 4°C and supernatant was aspirated (plaque-associated Aβ). Aliquots of the supernatant were diluted into room temperature FA neutralization solution (1 M Tris base, 0.5 M Na2HPO4, 0.05% NaN3). Aliquots samples were used in duplicate for protein content measurement by the method of Bradford; residual samples were stored at -80°C until subjected in duplicate in appropriate pre-dilution to Aβ42 ELISA as recommended by the vendor (Life Technologies). Samples of wild type mice served as specificity control for the ELISA (values obtained for hippocampal tissue were below the lowest standard and for cortical tissue 0 ng/g tissue were measured). Measurements were used for calculation of ng Aβ42/g tissue wet weight and normalized to content obtained for the soluble peptide amount in the cortex.

The BDNF ELISA was performed according to the manufacturer’s instructions (BosterBio). As no recommendations were given for tissue homogenates, we diluted samples with dilution buffer 1:8 and thereby obtained signals in the range of the standard.

Western Blotting

Brains were dissected under dim light after trans-cardiac perfusion of the animals with PBS, left hemisphere immediately stored in preservation buffer (10mM NEM; 2.5 mM EDTA in PBS), and shock frozen in liquid nitrogen. Samples were stored at -80°C until further use. Tissue was dissected on chilled metal plates to obtain visual cortex. Visual cortex parts were homogenized in preservation buffer supplemented with protease inhibitor cocktail (Roche) in a tissue lyzer (5min 50 Hz; Qiagen). Dilutions of tissue homogenates were used for protein determination (Nanoquant Reagent, Roth) and 17.5 (nNOS, NFκB p65, GluR1, GFAP) or 50 µg (IbaI) of protein separated on 10% SDS polyacrylamide gels. After transfer to nitrocellulose membrane, amount of selected markers was assessed by using appropriate primary antibodies and secondary antibodies labelled with horse radish peroxidase (ThermoScientific) with a chemiluminescent substrate (ThermoScientific) and a CCD Camera (Raytest) (for vendors of primary antibodies and dilutions see Suppl. Table 2). Gapdh served as a loading control and all densitometric values (AIDA) were normalized to data obtained for the respective Gapdh signal.

**NO measurement**

Dilutions of homogenates (description of preparation in Western Blot section) were mixed under dim light with Griess reagent (Enzo Life Science), incubated for 10 min in darkness and then measured at 540 nm. Values were normalized to protein content of homogenates.

All figure subpanels are outputs of the results obtained in Matlab or Prism in the versions stated; Figures were created in Adobe Illustrator 2019.


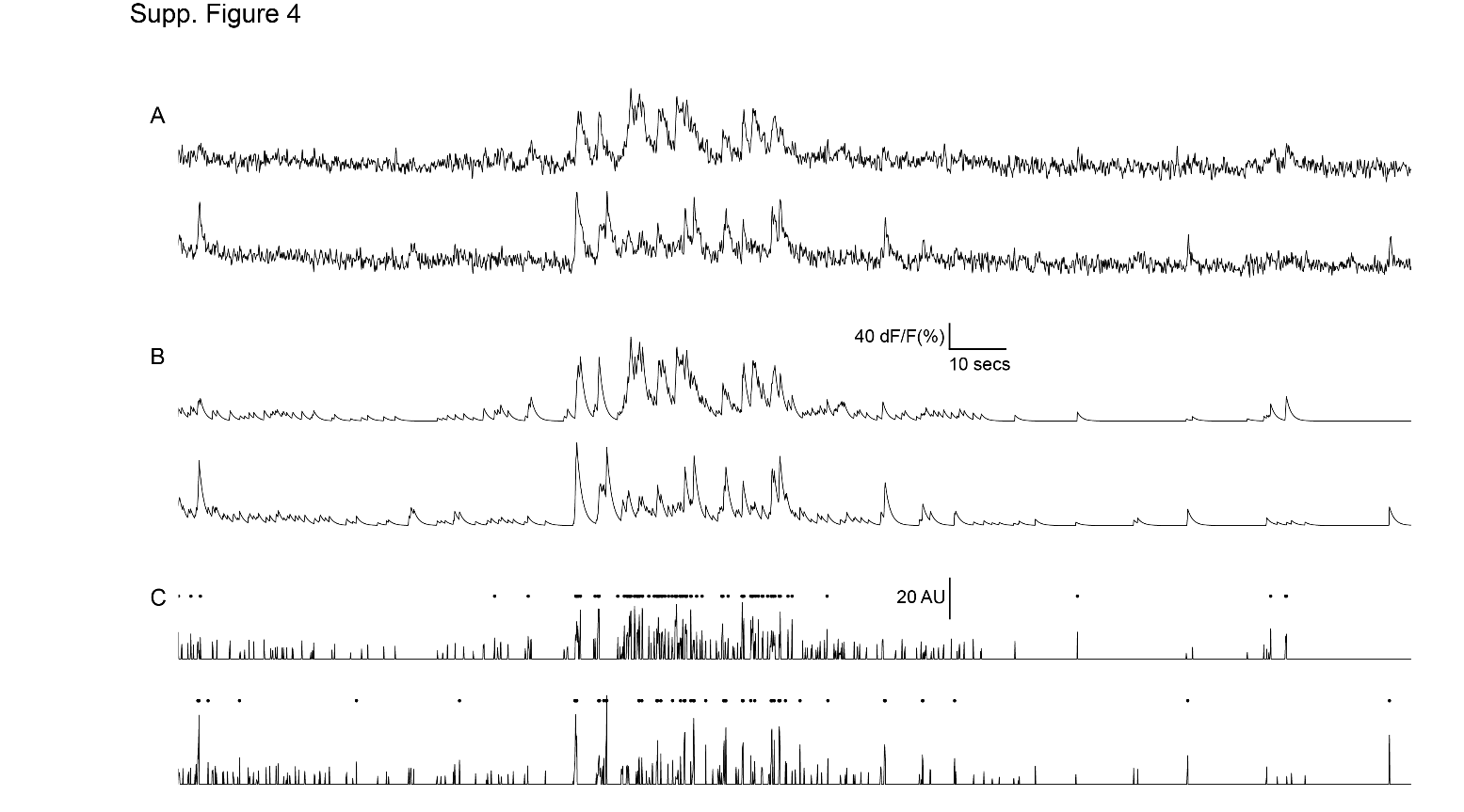


**Fig. S1. Sequential signal processing.** A. Raw calcium traces, signal is obtained from averaging fluorescence of every pixel within an ROI corresponding to a neuron’s soma. B. Denoised signals after processing with OASIS method. C. Deconvolved signals, black dots mark inferred action potentials.


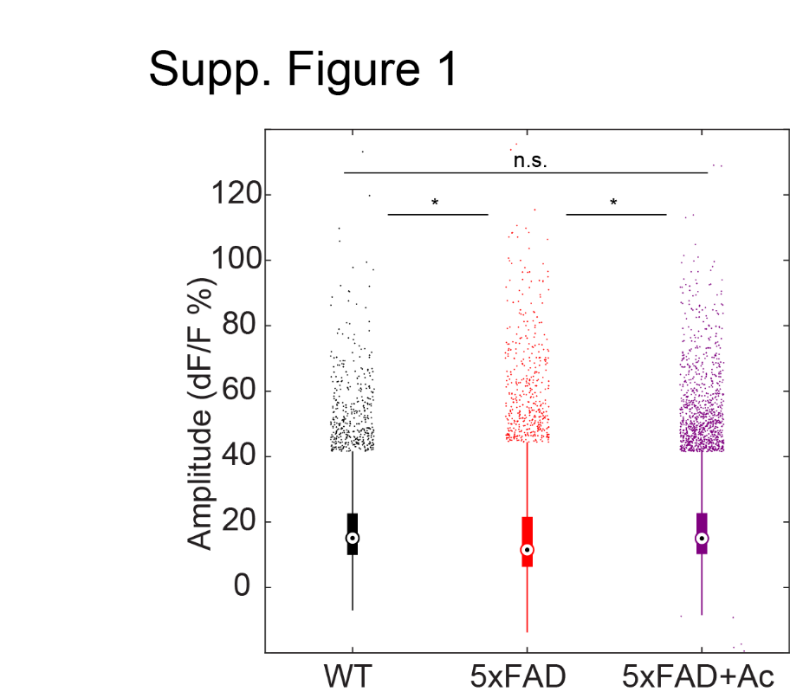


Fig. S2. Amplitude analysis of calcium transients. A. Distribution of amplitudes of the calcium transients. Measures from 5xFAD mice were significantly different from both, WT and AD+Ac, which were indistinguishable from each other. Kruskal-Wallis H Test (Chi-sq(2)=563.32, p=4.7589*E-123, mean ranks WT=17629, 5xFAD=14438, AD+Ac=17647).


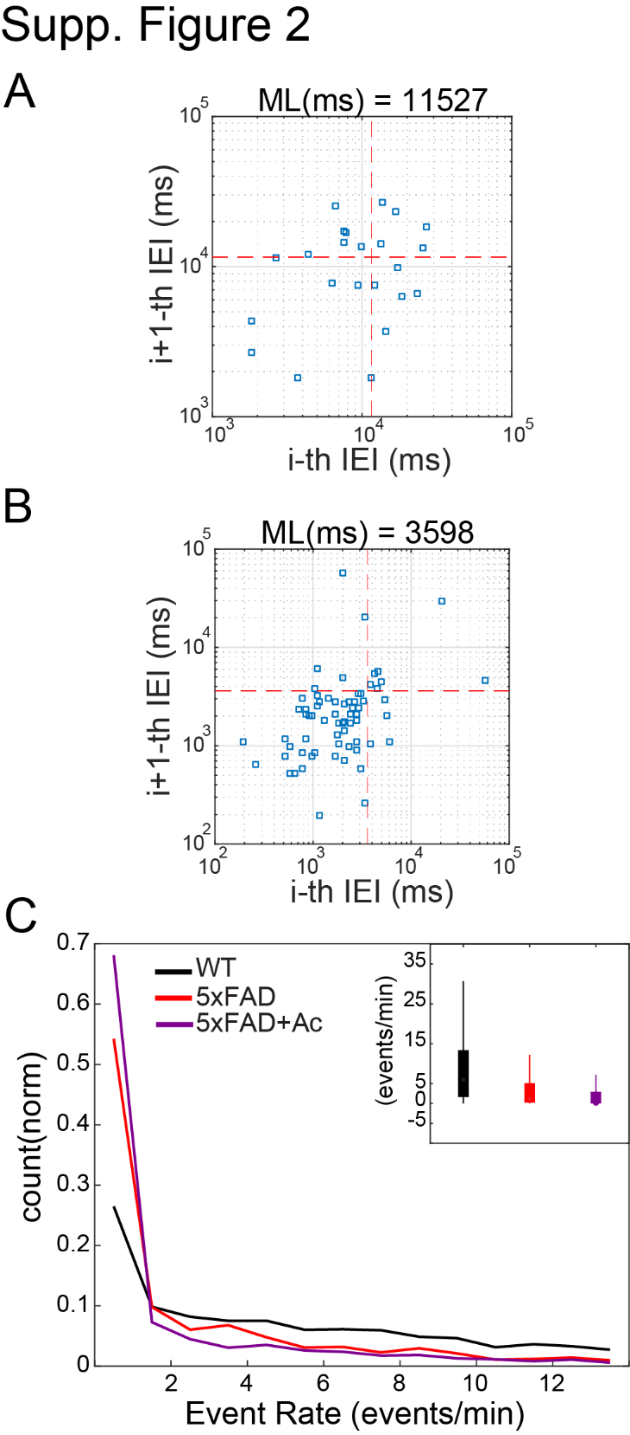


Fig. S3. Single-cell aberrant calcium transients and population event rate. A. Joint Inter Event Interval (JISI) histogram according to the data depicted in Figure 3 (A, E). Each square is a calcium transient event, and the abscissa and ordinate represent the intervals preceding and following a transient, respectively. B. JISI for 5xFAD neurons with aberrant transient patterns as depicted in Figure 3 (C, F). Note that given the bursting patterns present in the neuron, the majority of IEI are grouped in the lower left quadrant. C. Calcium transient event rate histogram for 3 experimental groups (5 animals each). Inset. Box plot summarizing distributions for 3 groups.


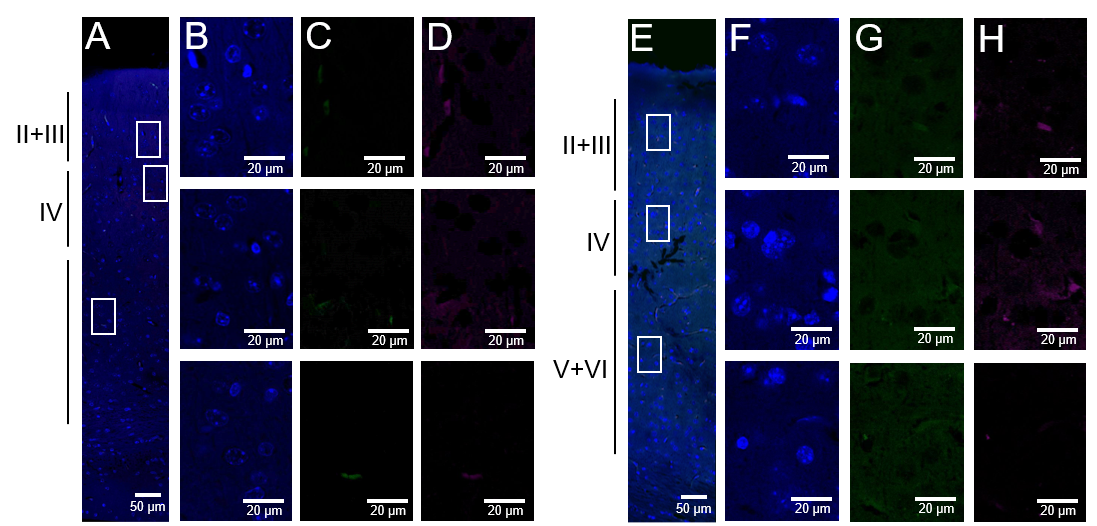


**Figure S4**. **Secondary antibody control.** A-D. Control staining for excitatory synapses. E-H. Control staining for inhibitory synapses. A. Overview of V1 area in 4 months old WT mice (green = Alexa488, magenta = Alexa568, blue = DAPI). Rectangles indicate sites of examples pictures shown in B-D. B. Example pictures of Layer II+III, Layer IV and Layer V+VI of the V1 region stained with DAPI. C+D. Example pictures of Layer II+II, Layer IV and Layer V+VI of the V1 region stained with Alexa488-conjugated anti-rabbit or Alexa568-conjugated anti-chicken respectively. E. Overview of V1 area in 4 months old WT mice (green = Alexa488, magenta = Alexa568, blue = DAPI). Rectangles indicate sites of examples pictures shown in F-H. F. Example pictures of Layer II+III, Layer IV and Layer V+VI of the V1 region stained with DAPI. G+H. Example pictures of Layer II+II, Layer IV and Layer V+VI of Alexa488-conjugated anti-rabbit and Alexa568-conjugated anti-guinea pig respectively. All antibodies are further specified in the Material and Methods section.


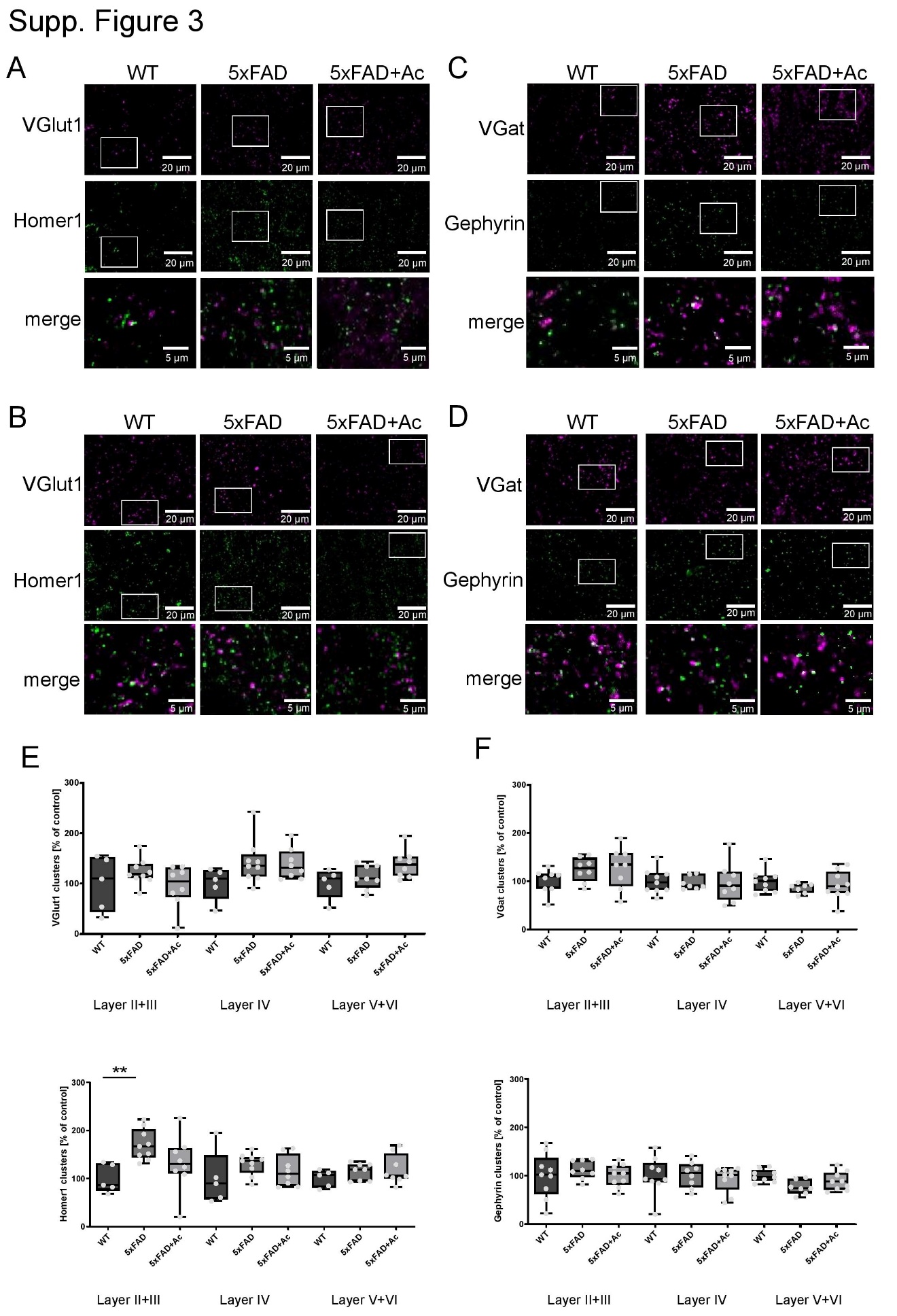


**Fig. S5. Number of excitatory and inhibitory synapses in Layer IV and V+VI**. A. Example pictures of pre- and postsynaptic clusters in WT, 5xFAD and 5xFAD+Ac mice. Shown is Layer IV. The 3rd row shows merged images which represent excitatory synapses (Selections are indicated in the pictures above). B. Example pictures of pre- and postsynaptic clusters in WT, 5xFAD and 5xFAD+Ac mice. Shown is Layer V+VI. The 3rd row shows merged images which represent excitatory synapses (Selections are indicated in the pictures above). C. Example pictures of pre- and postsynaptic clusters in WT, 5xFAD and 5xFAD+Ac mice. Shown is Layer IV. The 3rd row shows merged images which represent inhibitory synapses (Selections are indicated in the pictures above). D. Example pictures of pre- and postsynaptic clusters in WT, 5xFAD and 5xFAD+Ac mice. Shown is Layer V+VI. The 3rd row shows merged images which represent inhibitory synapses (Selections are indicated in the pictures above). E. Analysis of VGlut1 and Homer1 clusters in Layer II+III, Layer IV and Layer V+VI. Quantification shows that Homer1 clusters are selectively increased in Layer II+III of 5xFAD mice in comparison to WT or 5xFAD+Ac. F. Analysis of VGat and Gephyrin clusters in Layer II+III, Layer IV and Layer V+VI. Box plots indicate whiskers of the 5-95 percentile. P values were determined using one-way ANOVA (p < 0.0326) followed by Dunnett’s multiple comparisons test. Data are from 3 animals with 3 slices each.


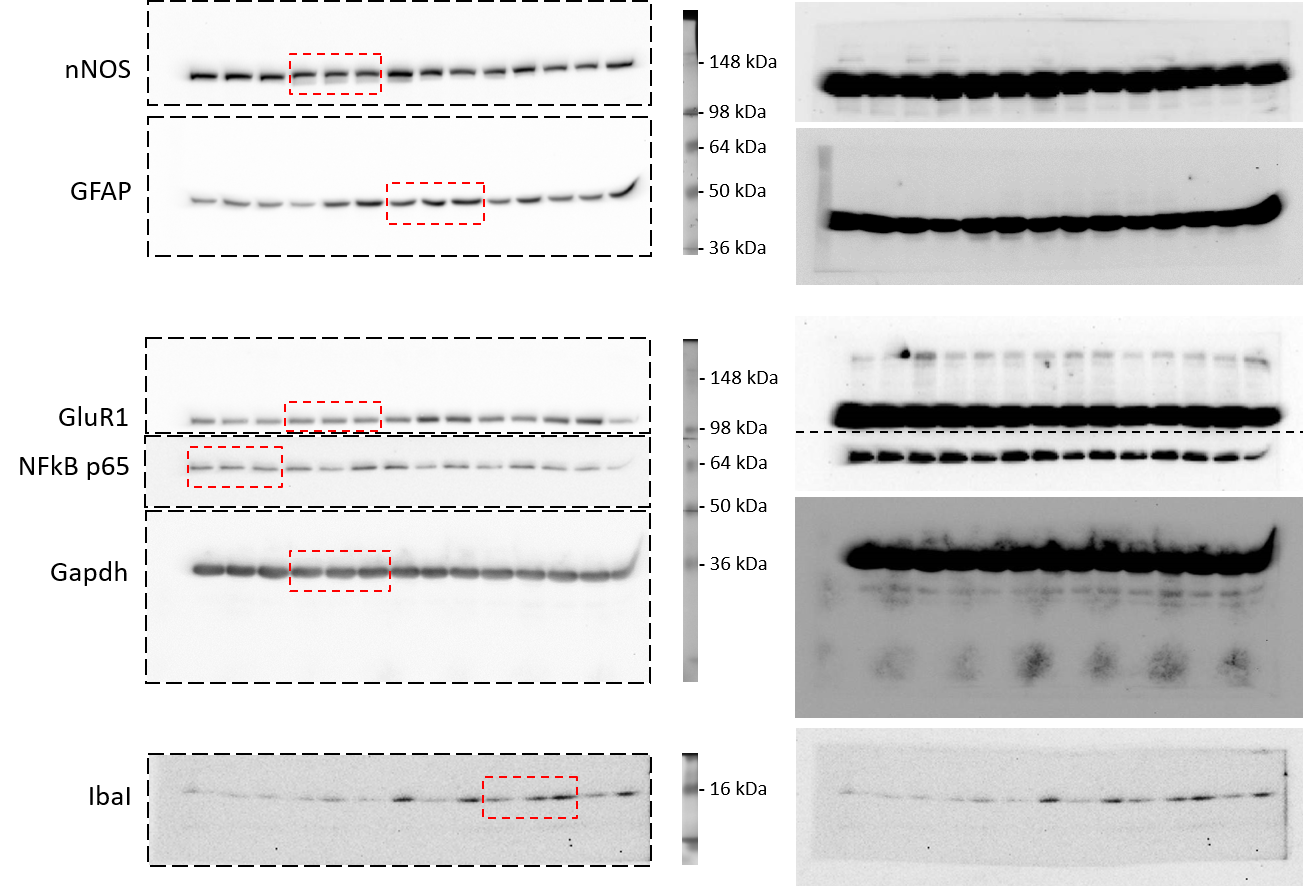


Figure S6. Full-length Western blots with corresponding marker bands. Pictures of uncropped Western blots with corresponding marker bands. Blots were cut in parts before the respective, indicated antibody incubation was conducted. The red dashed boxes illustrate which bands are used as examples in Figure 6. On the right, extremely overexposed images are shown to demonstrate borders of the respective membrane segments.

**Table S1. Overview of IEI parameters.**


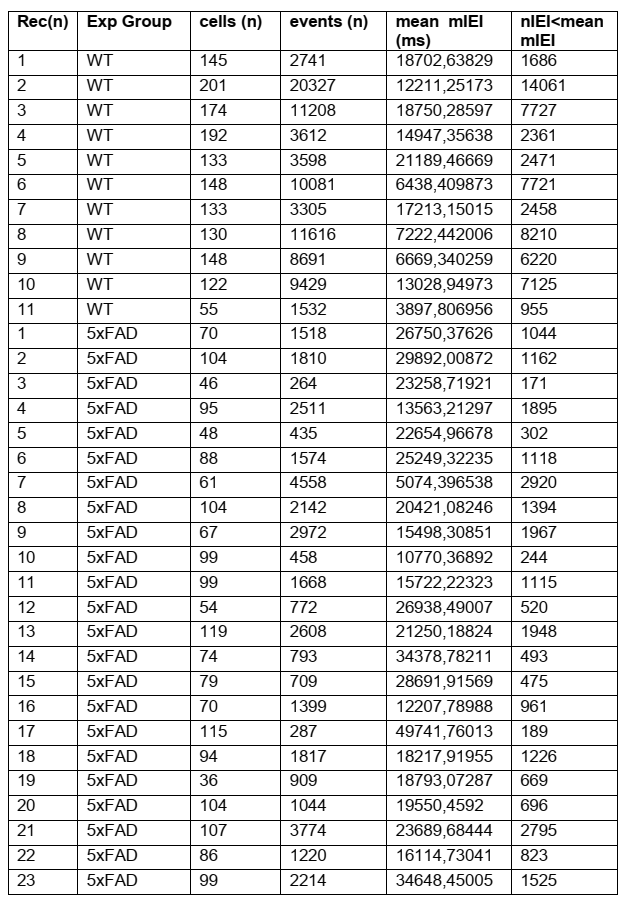


Table S2. Overview of Statistics for network measures derived from graph theory.


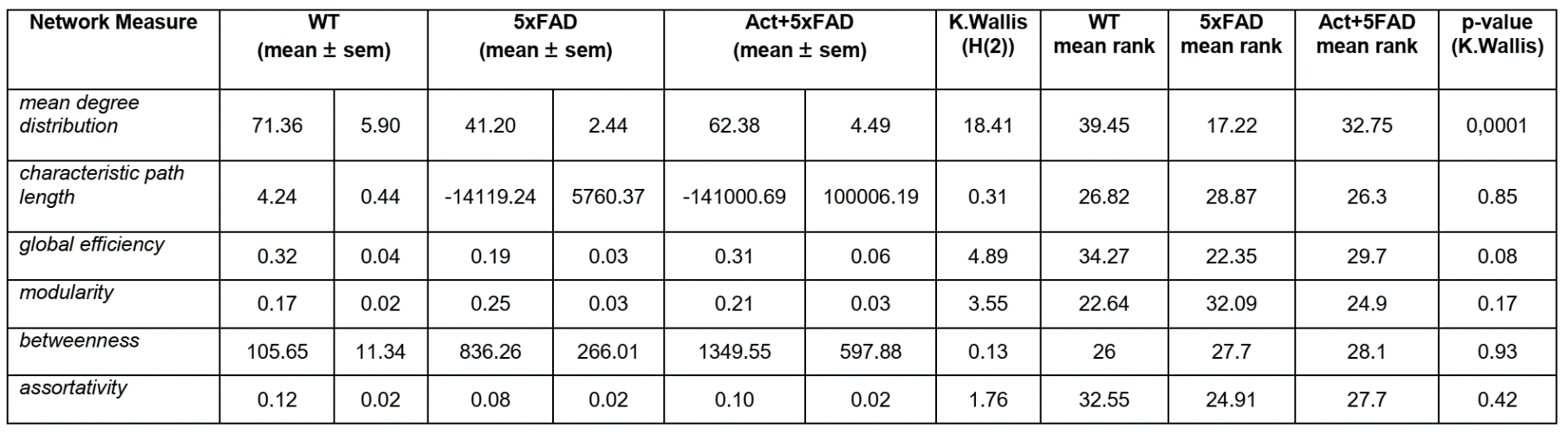


**Table S3. Mean Pearson’s coefficients per timelapse.**


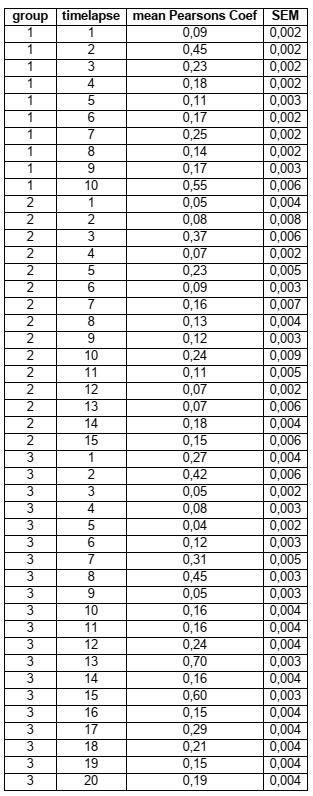


**Table S4. List of antibodies used in Western Blotting.**


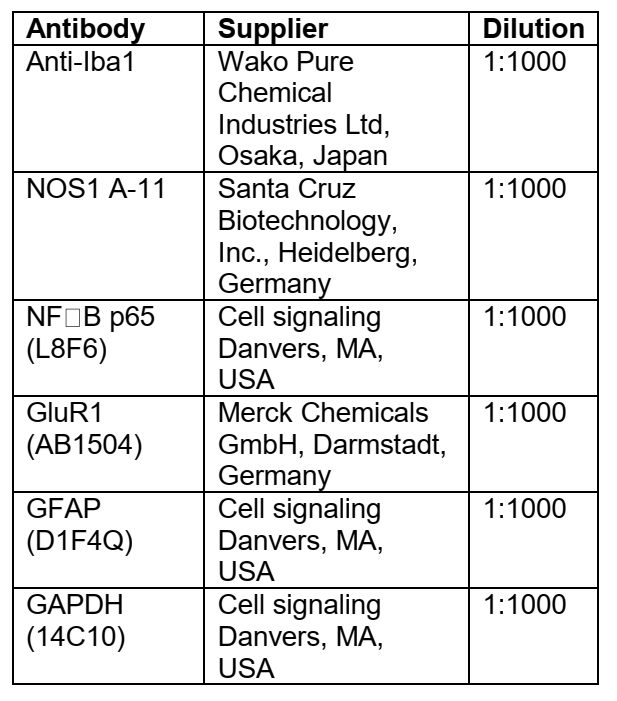


**SI References**

1 Brandscheid, C. *et al.* Altered Gut Microbiome Composition and Tryptic Activity of the 5xFAD Alzheimer's Mouse Model. *J Alzheimers Dis* **56**, 775-788, doi:10.3233/JAD-160926 (2017).

2 Paxinos, G. & Franklin, K. B. J. *Paxinos and Franklin's the mouse brain in stereotaxic coordinates*. 4th edn, (Boston : Elsevier/Academic Press, 2013).

3 Garaschuk, O., Milos, R. I. & Konnerth, A. Targeted bulk-loading of fluorescent indicators for two-photon brain imaging in vivo. *Nat Protoc* **1**, 380-386, doi:10.1038/nprot.2006.58 (2006).

4 Nimmerjahn, A., Kirchhoff, F., Kerr, J. N. & Helmchen, F. Sulforhodamine 101 as a specific marker of astroglia in the neocortex in vivo. *Nat Methods* **1**, 31-37, doi:10.1038/nmeth706 (2004).

5 Garaschuk, O. *et al.* Optical monitoring of brain function in vivo: from neurons to networks. *Pflugers Arch* **453**, 385-396, doi:10.1007/s00424-006-0150-x (2006).

6 Grienberger, C. *et al.* Sound-evoked network calcium transients in mouse auditory cortex in vivo. *J Physiol* **590**, 899-918, doi:10.1113/jphysiol.2011.222513 (2012).

7 Nimmerjahn, A. & Helmchen, F. In vivo labeling of cortical astrocytes with sulforhodamine 101 (SR101). *Cold Spring Harb Protoc* **2012**, 326-334, doi:10.1101/pdb.prot068155 (2012).

8 Friedrich, J., Zhou, P. & Paninski, L. Fast online deconvolution of calcium imaging data. *PLoS Comput Biol* **13**, e1005423, doi:10.1371/journal.pcbi.1005423 (2017).

9 Bullmore, E. & Sporns, O. Complex brain networks: graph theoretical analysis of structural and functional systems. *Nat Rev Neurosci* **10**, 186-198, doi:10.1038/nrn2575 (2009).

10 Amaral, L. A., Scala, A., Barthelemy, M. & Stanley, H. E. Classes of small-world networks. *Proc Natl Acad Sci U S A* **97**, 11149-11152, doi:10.1073/pnas.200327197 (2000).

11 Rubinov, M. & Sporns, O. Complex network measures of brain connectivity: uses and interpretations. *Neuroimage* **52**, 1059-1069, doi:10.1016/j.neuroimage.2009.10.003 (2010).

12 Achard, S. & Bullmore, E. Efficiency and cost of economical brain functional networks. *PLoS Comput Biol* **3**, e17, doi:10.1371/journal.pcbi.0030017 (2007).

13 Girvan, M. & Newman, M. E. Community structure in social and biological networks. *Proc Natl Acad Sci U S A* **99**, 7821-7826, doi:10.1073/pnas.122653799 (2002).

14 Morris, R. G., Anderson, E., Lynch, G. S. & Baudry, M. Selective impairment of learning and blockade of long-term potentiation by an N-methyl-D-aspartate receptor antagonist, AP5. *Nature* **319**, 774-776, doi:10.1038/319774a0 (1986).

15 Endres, K. *et al.* Transnasal delivery of human A-beta peptides elicits impaired learning and memory performance in wild type mice. *BMC Neurosci* **17**, 44, doi:10.1186/s12868-016-0280-9 (2016).

16 Schindelin, J. *et al.* Fiji: an open-source platform for biological-image analysis. *Nat Methods* **9**, 676-682, doi:10.1038/nmeth.2019 (2012).

17 Dzyubenko, E., Rozenberg, A., Hermann, D. M. & Faissner, A. Colocalization of synapse marker proteins evaluated by STED-microscopy reveals patterns of neuronal synapse distribution in vitro. *J Neurosci Methods* **273**, 149-159, doi:10.1016/j.jneumeth.2016.09.001 (2016).

18 Schmidt, S. D., Jiang, Y., Nixon, R. A. & Mathews, P. M. Tissue processing prior to protein analysis and amyloid-beta quantitation. *Methods Mol Biol* **299**, 267-278 (2005).
